# A Self-Report Measure of Diabetes Self-Management for Type 1 and Type 2 Diabetes: The Diabetes Self-Management Questionnaire-Revised (DSMQ-R) – Clinimetric Evidence From Five Studies

**DOI:** 10.3389/fcdhc.2021.823046

**Published:** 2022-01-13

**Authors:** Andreas Schmitt, Bernhard Kulzer, Dominic Ehrmann, Thomas Haak, Norbert Hermanns

**Affiliations:** ^1^ Research Institute of the Diabetes Academy Mergentheim (FIDAM), Diabetes Center Mergentheim (DZM), Bad Mergentheim, Germany; ^2^ German Center for Diabetes Research (DZD), Neuherberg, Germany; ^3^ Department of Clinical Psychology and Psychotherapy, University of Bamberg, Bamberg, Germany

**Keywords:** diabetes, treatment behavior, self-managament, health behavior, clinimetric, measurement instrument, questionnaire, evaluation

## Abstract

**Aims:**

Measurement tools to evaluate self-management behavior are useful for diabetes research and clinical practice. The Diabetes Self-Management Questionnaire (DSMQ) was introduced in 2013 and has become a widely used tool. This article presents a revised and updated version, DSMQ-R, and evaluates its properties in assessing self-management practices in type 1 diabetes (T1D) and type 2 diabetes (T2D).

**Methods:**

The DSMQ-R is a multidimensional questionnaire with 27 items regarding essential self-management practices for T1D and T2D (including diabetes-adjusted eating, glucose testing/monitoring, medication taking, physical activity and cooperation with the diabetes team). For the revised form, the original items were partially amended and the wording was updated; eleven items were newly added. The tool was applied as part of health-related surveys in five clinical studies (two cross-sectional, three prospective) including a total of 1,447 people with T1D and T2D. Using this data base, clinimetric properties were rigorously tested.

**Results:**

The analyses showed high internal and retest reliability coefficients for the total scale and moderate to high coefficients for the subscales. Reliability coefficients for scales including the new items were consistently higher. Correlations with convergent criteria and related variables supported validity. Responsiveness was supported by significant short to medium term changes in prospective studies. Significant associations with glycemic outcomes were observed for DSMQ-R-assessed medication taking, glucose monitoring and eating behaviors.

**Conclusions:**

The results support good clinimetric properties of the DSMQ-R. The tool can be useful for research and clinical practice and may facilitate the identification of improvable self-management practices in individuals.

## Introduction

Diabetes mellitus is a chronic metabolic disease characterized by elevated blood glucose levels due to absolute [type 1 diabetes (T1D)] or relative [type 2 diabetes (T2D)] insulin deficiency (1). The International Diabetes Federation estimates that 537 million adult people (20–79 years) are currently living with diabetes worldwide; the number is expected to rise to 643 million by 2030 (2). Diabetes care aims to help people with diabetes achieve near-normal glycemic levels in order to reduce the risk of long-term (e.g., vascular) complications of diabetes while avoiding acute metabolic risks and preserving best possible quality of life (3).

The key factor to achieving good glycemic levels is the person with diabetes’s self-management of their condition. People with diabetes may need to control carbohydrate intake via their selection of foods, adapt eating behaviors with regard to glycemic load, fats and healthy nutrition, manage blood glucose using glucose-lowering medications, monitor glucose levels using blood tests or sensors, engage in sufficient physical exercise (to optimize glycemia, manage weight or maintain good health) and arrange their activities around current glycemic levels and treatment requirements, as recommended by current guidelines (4–6). Where rapid acting insulin is used (to cover glucose rises after meals), estimating carbohydrate loads of the meals, dose-adjusting insulin doses and correcting elevated glucose levels are additional required practices of daily diabetes self-management.

Persistent or recurrent hyperglycemia increases the risk for developing serious long-term complications of diabetes such as diabetic retinopathy, neuropathy, nephropathy and foot syndrome; further, suboptimal glycemic management is associated with increased risks of acute metabolic complications such as severe hypoglycemia or severe hyperglycemia with the risk of ketoacidosis or hyperosmolar coma (7–9). Therefore, the adoption and maintenance of functional self-management behaviors to achieve good glycemic outcome is decisive for maintaining good health and preventing complications and morbidity (10). However, evidence supports that people with diabetes’ self-management practices and overall performance are often improvable (11, 12); this may be particularly true for people with comorbid mental conditions such as depression and diabetes-specific distress (13–15).

Since self-management is the decisive determinant of the course of diabetes, reflecting/monitoring relevant behaviors in individuals to identify areas of potential improvement and offer suitable education and support may be useful for routine clinical practice. The assessment and evaluation of diabetes self-management behaviors may be of particular interest in people with persistent suboptimum diabetes outcomes where possible problems and barriers are to be detected. Furthermore, measuring self-management may be required as part of research where facilitators and barriers to optimal diabetes care, including mental factors, shall be analyzed [e.g. (15, 16)] or effects of interventions (e.g., diabetes self-management education) are to be evaluated. Thus, suitable measurement tools are required.

Several systematic reviews of available measurement tools for diabetes self-management confirm that many different tools have been developed; however, most instruments have been applied in limited numbers of studies and the testing of measurement properties was often limited, with few scales meeting rigorous appraisal criteria, according to the reviewers’ conclusions (17–20). These problems may limit the available tools’ usability for research and practice.

In 2013, the Diabetes Self-Management Questionnaire [DSMQ (21)] was introduced to provide a multidimensional measure of diabetes self-management behaviors relevant for the control of glycemia in both major types of diabetes and to overcome limitations of contemporary questionnaires [e.g. (22)]. In direct comparisons, the DSMQ explained significantly more glycemic variation than an established standard self-care scale (21, 23). Since then, it has been translated into diverse languages and used in many studies, supporting its potential value for research and practice. A recent systematic review listed the DSMQ as one of only three scales on diabetes self-management which met the COSMIN (COnsensus-based Standards for the selection of health Measurement Instruments) guidelines for measurement tools that can be recommended for use and results obtained with can be trusted (20).

However, technological innovations such as continuous glucose monitoring and automatic insulin delivery have changed terms and expressions in diabetes care. Furthermore, a shift in diabetes-related language has taken place (24). Also, some specific self-management aspects should be better covered by the tool. For these reasons, a revision of the DSMQ was needed. The present article presents a revised and updated version of the tool and rigorous testing of its clinimetric properties and functions. Experiences with the tool’s use within five clinical studies provides a broad evidence base to inform about its characteristics and potentials.

## Materials and Methods

### Diabetes Self-Management Questionnaire (DSMQ)

The DSMQ is a multidimensional questionnaire consisting of self-descriptive statements from the person’s point of view (**Table 1**). Respondents are asked to reflect their self-management behaviors over the past weeks and rate to which extent each statement applies to them. An eight-week reference period was chosen to cover behaviors explaining present HbA_1c_; however, a shorter period (e.g., four weeks) might support the reflection of short-term changes, thus adaption of the instruction, where needed, might be considered. Responses are given on a four-point scale (from 0–’does not apply to me’ to 3–’applies to me very much’). Item scores are summed to scale scores reflecting the following specific activities: adjusting one’s diet towards diabetes (subscale ‘eating behavior’), taking medications consistently (subscale ‘medication taking’), testing/monitoring blood glucose or interstitial glucose (subscale ‘glucose monitoring’), being physically active to improve diabetes and health (subscale ‘physical activity’) and interacting with one’s diabetes-treating physician/healthcare professionals (subscale ‘cooperation with diabetes team’). A total score as a global measure of diabetes self-management can be calculated. Raw sum scores are transformed to a range from 0–10 for better interpretability and comparability (by dividing the raw sum score by the maximum possible sum of the scale [i.e., item number * 3] and multiplying with 10; details on scoring in **Supplementary Table 1**). The tool contains positively and negatively keyed items for greater validity and reliability (e.g., avoidance of one-sided, biased responses); negatively keyed items are reverse-scored before summing, thus higher scale scores reflect more optimal behavior. Since its introduction in 2013, the tool has been widely adopted and used for research and practice across countries and languages (**Supplementary Table 2**).

**Table 1:** Items of the original and revised versions of the DSMQ compared.

Original version with 16 items			Revised version with 27 items	
No.	Item	Level of revision^1^	No.	Item
1	I check my blood sugar levels with care and attention. (gm)^2^	≈	1	I check my glucose levels with care and attention. (gm)^2^
2	The food I choose to eat makes it easy to achieve optimal blood sugar levels. (eb)	≈	2	The foods I choose to eat make it easy for me to achieve good glucose levels. (eb)
3	I keep all doctors’ appointments recommended for my diabetes treatment. (cdt)	<	3	I regularly see the doctor (/diabetes specialist) regarding my diabetes. (cdt)
4	I take my diabetes medication (e.g. insulin, tablets) as prescribed. (mt)^3^	≈	4	I take my diabetes medication (e.g. insulin, tablets) consistently and reliably. (mt)^3^
5	Occasionally I eat lots of sweets or other foods rich in carbohydrates. (eb)^r^	≈	5	I occasionally eat large amounts of sweets or other foods rich in carbohydrates. (eb)^r^
6	I record my blood sugar levels regularly (or analyse the value chart with my blood glucose meter). (gm)^2^	<	6	I keep a diary/log of my glucose levels to inform and improve my diabetes management. (gm)^2^
7	I tend to avoid diabetes-related doctors’ appointments. (cdt)^r^	<	7	I tend to avoid seeing the doctor (/diabetes specialist) regarding my diabetes. (cdt)^r^
8	I do regular physical activity to achieve optimal blood sugar levels. (pa)	<	8	I am regularly physically active to improve my diabetes and health. (pa)
9	I strictly follow the dietary recommendations given by my doctor or diabetes specialist. (eb)	<	9	I follow the current dietary recommendations for people with diabetes (e.g. given to me by my doctor or diabetes specialist). (eb)
10	I do not check my blood sugar levels frequently enough as would be required for achieving good blood glucose control. (gm)^2r^	≈	10	I do not check my glucose levels frequently enough for achieving good blood glucose control. (gm)^2r^
11	I avoid physical activity although it would improve my diabetes. (pa)^r^	≈	11	I avoid physical activity although it would be good for my diabetes. (pa)^r^
12	I tend to forget to take or skip my diabetes medication (e.g. insulin, tablets). (mt)^3r^	≈	12	I tend to forget or skip taking my diabetes medication (e.g. insulin, tablets). (mt)^3r^
13	Sometimes I have real ‘food binges’ (not triggered by hypoglycemia). (eb)^r^	=	13	Sometimes I have real ‘food binges’ (not triggered by hypoglycemia). (eb)^r^
14	Regarding my diabetes care, I should see my medical practitioner(s) more often. (cdt)^r^	<	14	Regarding my diabetes, I should see my doctor (/diabetes specialist) more often. (cdt)^r^
15	I tend to skip planned physical activity. (pa)^r^	<	15	I am less physically active than would be good for my diabetes. (pa)^r^
16	My diabetes self-care is poor. (ts)^r^	=	20	(see below)
	n/a	/	16	I could improve my diabetes self-care considerably. (ts)^r^
	n/a	/	17	I estimate the carbohydrate content of my meals/foods (to improve my diabetes control). (eb)
	n/a	/	18	I eat without regard to my diabetes. (eb)^r^
	n/a	/	19	I check and discuss my diabetes treatment with the doctor (/diabetes specialist) regularly. (cdt)
16	(see above)	=	20	My diabetes self-care is poor. (ts)^r^
	n/a	/	21	I check my glucose levels before each meal.*
	n/a	/	22	I adjust my insulin doses to the carbohydrate content of my meals.*
	n/a	/	23	I adjust the timing of my insulin injections to the start of my meals.*
	n/a	/	24	I adjust my insulin doses according to the current glucose levels and preceding or planned activities.*
	n/a	/	25	I correct elevated glucose levels consistently whenever necessary.*
	n/a	/	26	I carry fast carbohydrates to enable quick treatment of low blood glucose.*
	n/a	/	27	In case of low blood glucose, I take appropriate amounts of carbohydrates to avoid causing high blood glucose.*

In comparing the original and revised items, item differences are highlighted by underlining.

Item-subscale information: (eb)=‘eating behavior’; (mt)=‘medication taking’; (gm)=‘glucose monitoring’; (pa)=‘physical activity’; (cdt)=‘cooperation with diabetes team’; (ts)=item included in total scale only.

^1^Level of revision/amendment of DSMQ-R items: ‘=‘ item is unchanged; ‘≈’ item is only minimally/slightly revised and contentually equivalent to its previous version; ‘<‘ item wording is changed, but essential meaning remains/is comparable to its previous version; ‘/’ item is newly added, no related item present in original 16-item form.

^2^Item enables ticking “Glucose checking/monitoring is not required as a part of my self-care.”, for where applicable.

^3^Item enables ticking “Glucose-lowering medication is not required as a part of my self-care.”, for where applicable.

^r^Reverse-scored item.

*Optional item to be answered by people with intensive insulin treatment (i.e. multiple daily insulin injections) only.

#### Original Version

The original version of the DSMQ consists of 16 items (**Table 1**) which were developed and selected in a systematic, iterative process: A set of newly developed and qualitatively piloted items were initially tested on a sample of 110 people and successively excluded until only those with good properties remained (21). The resulting questionnaire was then administered to 261 people with T1D or T2D to evaluate measurement properties against a convergent standard measure; results supported reliability and validity (21). A subsequent study yielded further supportive evidence (23).

#### Revision

Reasons for the revision were: i) wording considered as improvable in single items, ii) findings suggesting limited reliability for the ‘cooperation with diabetes team’ subscale in some studies and iii) practices of dose-adjusting insulin injections and correcting glucose levels (where intensive insulin treatment applies) being insufficiently covered. The original scale was amended accordingly, that is: i) items were updated to conform with new technologies such as continuous glucose monitoring (CGM) and data management software; the potentially misleading term ‘blood sugar levels’ was replaced with ‘glucose levels’, referring to both blood and interstitial glucose; some items were revised to avoid compliance-oriented expressions (e.g., ‘strictly follow’ or ‘as prescribed’); ii) the ‘cooperation with diabetes team’ items were harmonized and one additional item was added to improve reliability; iii) seven items covering practices of intensive insulin treatment were added as an optional extra. Item-level amendments are given in detail in **Supplementary Table 3**; old and new items are compared in **Table 1**. In summary, two items remained unchanged, seven items were slightly revised and seven items were significantly altered but the essential meaning was kept (**Table 1**). The original item order was kept, except for item 16 which was repositioned as number 20. A total of eleven items were newly added, thereof four regarding general behaviors (no. 16–19) and seven (no. 21–27) regarding intensive insulin treatment practices specifically (e.g., adjusting insulin; correcting glucose levels), the latter given in a separate section with specific instruction.

The DSMQ-R thus contains a total of 27 items, 20 on general behaviors relevant for most people with diabetes and seven on specific insulin treatment behaviors. A total score is estimated using the 20 general items; where applicable, a 27-item total score including the optional items can be calculated. The subscale ‘eating behavior’ contains now six items and the subscale ‘cooperation with diabetes team’ four; ‘medication taking’, ‘glucose monitoring’ and ‘physical activity’ remain unchanged with two, three and three items, respectively; two of the 20 general items request global statements and are included in the total scale only (**Table 1**).

### Study Design and Data Collection

This evaluation of the DSMQ-R includes T1D and T2D. The analyzed data were acquired as part of five clinical studies, three cross-sectional, two prospective, conducted between 2015 and 2021. All studies were ethically approved and carried out in accordance with the Declaration of Helsinki. All participants provided written informed consent.

- *Study 1* was a multi-center, cross-sectional survey to evaluate person-reported outcome measures for diabetes, conducted in 2015–16; details of the study are reported elsewhere (25). Ethical approval was obtained from the Ethics Committee of the German Psychological Society (file no. NH 032015). *N*=606 participants were surveyed using questionnaires including the DSMQ-R, DAS, PAID-5, PHQ-9, DTSQ (explained below); 606 people participated; data of *n*=588 (56.6% T1D) could be used for this evaluation.
- *Study 2* (‘Depression and Diabetes Control Trial’) was a randomized controlled trial testing a diabetes-specific treatment program for people with depressive symptoms (CES-D ≥16) and hyperglycemia (HbA_1c_ >7.5%/59 mmol/mol) against diabetes care as usual. It was approved by the Ethics Committee of the State Medical Chamber of Baden-Wuerttemberg (file no. F-2015-056) and is registered at ClinicalTrials.gov (ID no. NCT02675257). Participants were enrolled from 1/2016–3/2017. The first follow-up (FU) assessment after six months was used for retest analysis in this evaluation. Questionnaire assessments included the DSMQ-R, SDSCA, PAID, DDS, DAS and PHQ-9 (below). HbA_1c_ was assessed in a central laboratory. *N*=213 were enrolled and 198 (66.2% T1D) provided suitable data for this evaluation.
- *Study 3:* a cross-sectional FU survey of the ‘DIAMOS’ and ‘ECCE HOMO’ trial participants was conducted in 2017–18, on average five years after participating in the original trials. Participants had been enrolled using equivalent inclusion and exclusion criteria, enabling aggregation to one cohort; all had elevated depressive symptoms (CES-D ≥16) at baseline [study details accessible elsewhere (26–28)]. The FU was approved by the Ethics Committee of the State Medical Chamber of Baden-Wuerttemberg (file no. F-2017-071). A total of 323 people (68.1% of the total cohort) could be followed up using questionnaires including the DSMQ-R, PAID, DDS, DAS and PHQ-9; HbA_1c_ was estimated. *N*=298 people (64.0% T1D) provided sufficient data for this evaluation.
- *Study 4* (‘DIA-LINK1’) was a prospective observational study analyzing links between mental health and glycemic outcomes in T1D (ClinicalTrials.gov ID no. NCT03811132); participants were enrolled from 3/2019–3/2020 and followed over three months. Measurements comprised repeated surveys (including DSMQ-R, PAID, T1-DDS, CES-D), HbA_1c_ estimation, 17-day ecological momentary assessment (EMA) with daily diabetes-related questions and 4-week continuous glucose monitoring (CGM) (29). The study was approved by the Ethics Committee of the German Psychological Society (file no. NH 082018). *N*=203 participants were enrolled.
- *Study 5:* the ‘DIA-LINK2’ study (2020–21) is a prospective observational study regarding mental health and glycemia with the same design as DIA-LINK1 but regarding T2D (ClinicalTrials.gov ID no. NCT04438018). Ethical approval was obtained from the Ethics Committee of the German Psychological Society (file no. HermannsNorbert2020-03-05AM). A total of 190 people with T2D have been enrolled, and *n*=180 provided suitable data for this evaluation.

### Variables and Measurements

Besides the DSMQ-R, the following variables were assessed as part of the studies:

*Glycemic outcome:* Glycated hemoglobin (HbA_1c_) was estimated from venous blood samples taken at the same time as the questionnaire assessments in all studies. HbA_1c_ was usually estimated in a central laboratory (at the Diabetes Center Mergentheim) using high performance liquid chromatography (performed with the Bio-Rad Variant II Turbo analyzer in studies 2 and 3 and the Tosoh Automated Glycohemoglobin Analyzer HLC-723G11 in studies 4 and 5), meeting IFCC standard [laboratory normal range 4.3–6.1% (24–43 mmol/mol)]; study 1 included four different laboratory cites.

Study 4 additionally assessed glycemic levels over four weeks using intermittently scanned CGM. The following CGM-derived parameters were calculated: mean sensor glucose (in mg/dl), time in range (% values between 70–180 mg/dl, 3.9–10 mmol/l), time below range (% values <70 mg/dl, <3.9 mmol/l), time above range (% values >180 mg/dl, >10 mmol/l), and glucose variability [coefficient of variation (CV)].

*Diabetes self-care activities:* The 10-item Summary of Diabetes Self-Care Activities Measure [SDSCA (22, 30)] was applied as a convergent measure of diabetes self-management in study 2. The tool requests on how many days of the past week the person engaged in healthy eating, exercising, blood sugar testing and foot care. Responses are averaged to scales (e.g., Diet, Exercise, Blood Sugar Testing) with scores ranging from 0–7 and higher values reflecting more frequent activity.

*Diabetes distress and diabetes-specific problems:* The 20-item Problem Areas in Diabetes Scale (PAID) measuring diabetes-related distress (31) was applied in all studies. The questionnaire requests ratings of diabetes-specific emotional problems on a five-point scale (0–’not a problem’ to 4–’serious problem’). The item scores are summed and transformed to a total score ranging from 0–100; higher scores reflect higher distress; scores ≥40 suggest meaningful distress (32). In study 1, the 5-item short form [PAID-5 (33)] was used.

In studies 2–5, the Diabetes Distress Scale [DDS (34)] or T1-Diabetes Distress Scale [T1-DDS (35)] was administered in addition to the PAID. The DDS/T1-DDS items address a range of diabetes-specific problems; however, it also includes items and scales whose relations to the construct of diabetes distress have been questioned (14, 32, 36). Therefore, we did not estimate a total score but rather selected specific items whose contents regarding self-management-related problems could be used for the correlation analysis (i.e., DDS items 6, 8 and 12 on ‘not testing blood sugars frequently enough’, ‘often failing with diabetes routine’ and ‘not sticking closely enough to a good meal plan’, and T1-DDS items 2, 8, 12, 23 and 28 on ‘not eating as carefully as one should’, ‘not taking as much insulin as one should’, ‘not checking blood glucose as often as one should’, ‘eating being out of control’ and ‘not giving diabetes as much attention as one should’); these aspects were assessed as convergent criteria for corresponding DSMQ-R scales. Items regarding doctor-related problems (i.e., DDS item 15 on ‘not having a doctor who one can see regularly about diabetes’ and T1-DDS items 7 and 18, ‘can’t tell diabetes doctor what is really on my mind’, ‘diabetes doctor doesn’t really understand what it’s like to have diabetes’) were used for correlation with the DSMQ-R scale ‘cooperation with diabetes team’. Responses in the DDS/T1-DDS are given on a six-point scale (1–’not a problem’ to 6–’a very serious problem’), thus higher scores reflect greater problems.

*Diabetes acceptance*, a measure of psychological adjustment to living with diabetes, was assessed using the Diabetes Acceptance Scale (DAS); in studies 1–3, the full 20-item version was used, in studies 4–5, the 10-item short form (25). The items request aspects of acceptance and integration (e.g., ‘I accept diabetes as part of my life’) versus avoidance, neglect and demotivation (e.g., ‘I avoid dealing with topics related to diabetes’). Responses are given on a four-point scale (0–’never true for me’ to 3–’always true for me’). Item scores are summed so that higher scores reflect higher acceptance (range 0–60). Higher acceptance scores have been associated with more optimal self-management (25, 37). Besides the total score, items specifically related to treatment motivation (e.g., ‘I have difficulties to motivate myself to perform good diabetes self-care’) and treatment neglect (e.g., ‘I neglect diabetes self-care because I want to avoid topics related to diabetes’) were aggregated to subscales (Cronbach’s *α*=0.71 and 0.83, respectively).

*Diabetes treatment satisfaction* was measured using the Diabetes Treatment Satisfaction Questionnaire (DTSQ) in study 1, including six satisfaction-related items and a 7-point scale (0–’very dissatisfied’ to 6–’very satisfied’). Items are summed to a total score from 0–64; higher scores reflect higher satisfaction (38). Higher treatment satisfaction was expected to be associated with more optimal treatment behavior (DSMQ-R).

*Depressive symptoms* were assessed in all studies due to their high prevalence in diabetes as well as the studies focusing on depression and mental health. Studies included either the Patient Health Questionnaire-9 (PHQ-9) or the Center for Epidemiologic Studies Depression Scale (CES-D); both have excellent properties (39). The PHQ-9 assesses the nine symptoms of major depression according to DSM-5 during the past two weeks. Responses are given on a four-point scale (0–’not at all’ to 3–’nearly every day’). Total score range is 0–27; higher scores indicate more symptoms. The CES-D assesses 20 depressive symptoms during the past week; responses are given on a four-point scale (0–’rarely or none of the time’ to 3–’most or all of the time’), resulting in a total score from 0–60 (higher scores=more symptoms). Depressive symptoms have been consistently associated with less optimal self-management across behaviors [e.g. (13)].

*Daily diabetes problems/burdens:* The DIA-LINK studies included a smartphone-based EMA with daily diabetes-related questions over 17 days (29). Items constituting likely correlates of the DSMQ-R were used as convergent criteria (e.g., ‘How much have you felt guilty when neglecting your diabetes treatment today?’; full item details in **Supplementary Table 4**). Responses were given on a scale from 0–’not at all’ to 10–’very much’. Daily responses were averaged.

*Demographic and person-related variables* comprised sex, age, BMI, diabetes type, diabetes duration and treatment regimen. Long-term and acute complications of diabetes (study 1) were based on medical examinations, laboratory assessments and interviews (assessed were diabetic retinopathy, neuropathy, nephropathy, foot syndrome; treated ketoacidosis, past 12 months). Mean numbers of daily insulin injections (where applicable) and daily glucose tests or scans/readings as well as frequencies of diabetologist visits per past six months were assessed in face-to-face interviews.

### Statistical Analyses

Statistical analyses were performed using SPSS 26.0.0 (IBM SPSS Statistics). *P* values < 0.05 (two-tailed) were considered to indicate statistical significance. For the DSMQ-R, total and subscale scores were calculated as per scoring instruction (**Supplementary Table 1**) with scores ranging between 0 and 10. Negatively-keyed items were reverse-scored so that higher scale scores suggest more optimal behavior. Where applicable, a 27-item total score was calculated in addition to the 20-item total; yet the optional items were not included in subscale scores to warrant comparisons of scores between subgroups. Measurement functions were analyzed according to clinimetric criteria (40). *Internal reliability* was analyzed using Cronbach’s *α*; since potential preference of McDonald’s *ω* over *α* has been discussed (41), *ω* was additionally estimated [using Hayes’ OMEGA macro for SPSS (41)]. *Reproducibility* was tested using retest correlations in the prospective studies. *Construct validity* was evaluated *via* correlations with convergent measures and related variables to develop a nomological network. Since adjusting eating behaviors towards diabetes, taking medications consistently and checking glucose levels regularly can be expected to result in better glycemic levels, associations between the corresponding DSMQ-R scales and glycemic outcomes were analyzed as indicators of validity. Similarly, associations with acute and long-term complications were assessed in study 1. Further, associations between the DSMQ-R scales and convergent measures of self-care activities, treatment satisfaction, treatment motivation and neglect as well as diabetes acceptance, diabetes distress and depressive symptoms were analyzed. *Structural validity* was assessed using confirmatory factor analyses (AMOS 26.0.0, IBM SPSS Statistics). Model fit was evaluated according to Comparative Fit Index (CFI) ≥ 0.95, Tucker Lewis Index (TLI) ≥ 0.95, Standardized Root Mean Square Residual (SRMR) ≤ 0.08 and Root Mean Square Error of Approximation (RMSEA) ≤ 0.06. *Responsiveness*, the ability to detect change, was assessed via changes of the DSMQ-R scales in prospective studies, given as Cohen’s *d*. Where applicable, changes were compared between treatment groups (i.e., study 2, with participants randomized to either depression treatment or diabetes care as usual).

## Results

### Sample Characteristics

The sample characteristics are given in **Table 2**. Studies 1–3 had mixed samples including people with T1D and T2D (T1D being overrepresented in line with secondary and tertiary care enrolment), study 4 and 5 assessed only T1D or T2D, respectively. Sample sizes varied between 180 and 588. Study 1 contained a more general sample, whereas other studies overrepresented people with specific mental aspects: study 2 contained people with current depressive symptoms, study 3 contained people with a history of depressive symptoms and studies 4 and 5 included majorities with either depressive symptoms or diabetes distress. All samples had a wide age range with a mean age between 45 and 53 years, except for study 4 (T1D only) whose sample’s mean age was 39 years. The mean diabetes duration reflected relatively long-standing diabetes throughout. HbA_1c_ levels were generally elevated with mean values around 7.8 to 9.3% (62 to 78 mmol/mol) across the studies.

**Table 2:** Study sample characteristics and normative data and reliability indices for the DSMQ-R scales by study and diabetes type.

Studies	*n*	Sample characteristics	DSMQ-R scales	*N* items (item numbers)	Mean ±SD scale scores		Internal reliability coefficients (Cronbach’s *α* [McDonald’s *ω*])		Test-retest reliability coefficients (Pearson’s *r*)	
					T1D	T2D	T1D	T2D	T1D	T2D
Study 1: Cross-sectional questionnaire study (2015–16)	588	Adults with T1D or T2D Ø age: 49.5 ±15.2 (range 18–82) years 55.6% women *n*=333 with T1D *n*=255 with T2D (thereof 87 with MDI^1^) Ø DM duration: 15.7 ±11.2 years Ø HbA_1c_: 8.2% ±1.6 (66 mmol/mol ±18) Ø PHQ-9 depression score: 7.7 ±5.6	20-item total score	20 (1–20)	6.4±2.0	6.4 ±1.5	0.92 (0.92)	0.84 (0.84)	n/a	n/a
			27-item total score^1^	27 (1–27)	6.4 ±2.1	6.6 ±1.6^1^	0.94 (0.95)	0.90 (0.90)^1^	n/a	n/a
			Eating behavior	6 (2,5^r^,9,13^r^,17,18^r^)	5.6 ±2.2	5.5 ±2.0	0.81 (0.81)	0.75 (0.76)	n/a	n/a
			Medication taking	2 (4,12^r^)	8.1 ±2.8	8.7 ±2.0	0.84 (n/a)	0.66 (n/a)	n/a	n/a
			Glucose monitoring	3 (1,6,10^r^)	6.4 ±3.2	6.9 ±2.9	0.82 (0.83)	0.78 (0.79)	n/a	n/a
			Physical activity	3 (8,11^r^,15^r^)	5.8 ±2.9	4.8 ±2.6	0.84 (0.84)	0.72 (0.72)	n/a	n/a
			Cooperation with diabetes team	4 (3,7^r^,14^r^,19)	7.8 ±2.3	8.0 ±1.8	0.78 (0.78)	0.56 (0.56)	n/a	n/a
Study 2: ‘Depression and Diabetes Control Trial’ (2016–17), prospective randomized trial, retest after six months	198	Adults with T1D or T2D with elevated depressive symptoms Ø age: 45.4 ±13.6 (range 18–69) years 57.6% women *n*=131 with T1D *n*=67 with T2D (thereof 40 with MDI^1^) Ø DM duration: 15.8 ±9.6 years Ø HbA_1c_: 9.3% ±1.4 (78 mmol/mol ±15) Ø CES-D depression score: 24.0 ±11.0	20-item total score	20 (1–20)	5.4 ±2.1	5.4 ±1.7	0.91 (0.91)	0.87 (0.86)	0.62	0.48
			27-item total score^1^	27 (1–27)	5.4 ±2.0	5.7 ±1.7^1^	0.93 (0.93)	0.91 (0.90)^1^	0.61	0.62^1^
			Eating behavior	6 (2,5^r^,9,13^r^,17,18^r^)	4.7 ±2.2	4.2 ±2.1	0.78 (0.79)	0.77 (0.76)	0.57	0.46
			Medication taking	2 (4,12^r^)	7.4 ±2.9	7.9 ±2.5	0.80 (n/a)	0.81 (n/a)	0.62	0.54
			Glucose monitoring	3 (1,6,10^r^)	4.9 ±3.3	5.4 ±3.6	0.81 (0.82)	0.89 (0.90)	0.53	0.73
			Physical activity	3 (8,11^r^,15^r^)	5.1 ±3.3	4.3 ±2.7	0.87 (0.87)	0.76 (0.77)	0.68	0.23
			Cooperation with diabetes team	4 (3,7^r^,14^r^,19)	7.0 ±2.8	7.8 ±2.3	0.85 (0.85)	0.73 (0.73)	0.46	0.54
Study 3: Five-year FU of the DIAMOS-ECCE HOMO cohort (2017–18), cross-sectional study	298	Adults with T1D or T2D and a history of depressive symptoms Ø age: 52.3 ±13.3 (range 23–77) years 57.7% women *n*=191 with T1D *n*=107 with T2D (thereof 79 with MDI^1^) Ø DM duration: 21.6 ±11.1 years Ø HbA_1c_: 7.8% ±1.1 (62 mmol/mol ±12) Ø PHQ-9 depression score: 8.2 ±5.0	20-item total score	20 (1–20)	6.7 ±1.6	6.3 ±1.7	0.88 (0.88)	0.89 (0.89)	n/a	n/a
			27-item total score^1^	27 (1–27)	6.9 ±1.6	6.5 ±1.6^1^	0.91 (0.91)	0.91 (0.91)^1^	n/a	n/a
			Eating behavior	6 (2,5^r^,9,13^r^,17,18^r^)	5.8 ±1.8	5.3 ±2.1	0.72 (0.73)	0.78 (0.80)	n/a	n/a
			Medication taking	2 (4,12^r^)	8.3 ±2.2	8.6 ±2.4	0.73 (n/a)	0.72 (n/a)	n/a	n/a
			Glucose monitoring	3 (1,6,10^r^)	7.0 ±2.6	6.6 ±3.0	0.72 (0.74)	0.80 (0.80)	n/a	n/a
			Physical activity	3 (8,11^r^,15^r^)	6.0 ±3.0	4.5 ±2.8	0.88 (0.88)	0.86 (0.87)	n/a	n/a
			Cooperation with diabetes team	4 (3,7^r^,14^r^,19)	8.4 ±2.1	8.2 ±1.9	0.80 (0.80)	0.65 (0.66)	n/a	n/a
Study 4: ‘DIA-LINK1’ (2019–20), prospective observational study, retest after three months	203	Adults with T1D, majority with diabetes distress and/or depressive symptoms Ø age: 38.6 ±12.8 (range 18–69) years 58.1% women 100% with T1D Ø DM duration: 18.5 ±11.7 years Ø HbA_1c_: 8.7% ±1.9 (71 mmol/mol ±21) Ø CES-D depression score: 21.3 ±11.4 Ø PAID diabetes distress score: 40.1 ±18.8	20-item total score	20 (1–20)	5.8 ±1.8	n/a	0.88 (0.88)	n/a	0.66	n/a
			27-item total score	27 (1–27)	6.0 ±1.7	n/a	0.91 (0.91)	n/a	0.64	n/a
			Eating behavior	6 (2,5^r^,9,13^r^,17,18^r^)	4.6 ±2.1	n/a	0.74 (0.75)	n/a	0.66	n/a
			Medication taking	2 (4,12^r^)	7.4 ±2.6	n/a	0.78 (n/a)	n/a	0.53	n/a
			Glucose monitoring	3 (1,6,10^r^)	5.8 ±2.8	n/a	0.69 (0.72)	n/a	0.55	n/a
			Physical activity	3 (8,11^r^,15^r^)	5.5 ±3.2	n/a	0.87 (0.87)	n/a	0.69	n/a
			Cooperation with diabetes team	4 (3,7^r^,14^r^,19)	7.9 ±2.4	n/a	0.83 (0.83)	n/a	0.59	n/a
Study 5: ‘DIA-LINK2’ (2020–21), prospective observational study, retest after three months	180	Adults with T2D, majority with diabetes distress and/or depressive symptoms Ø age: 52.9 ±9.8 (range 23–70) years 38.9% women 100% with T2D (thereof 121 with MDI^1^) Ø DM duration: 12.1 ±8.0 years Ø HbA_1c_: 9.1% ±1.7 (76 mmol/mol ±19) Ø CES-D depression score: 22.2 ±12.1 Ø PAID diabetes distress score: 41.7 ±19.1	20-item total score	20 (1–20)	n/a	5.4 ±1.8	n/a	0.89 (0.89)	n/a	0.58
			27-item total score^1^	27 (1–27)	n/a	5.7 ±1.8^1^	n/a	0.92 (0.92)^1^	n/a	0.57^1^
			Eating behavior	6 (2,5^r^,9,13^r^,17,18^r^)	n/a	4.3 ±2.2	n/a	0.80 (0.80)	n/a	0.56
			Medication taking	2 (4,12^r^)	n/a	7.5 ±2.9	n/a	0.82 (n/a)	n/a	0.55
			Glucose monitoring	3 (1,6,10^r^)	n/a	5.1 ±3.1	n/a	0.79 (0.80)	n/a	0.48
			Physical activity	3 (8,11^r^,15^r^)	n/a	4.2 ±3.1	n/a	0.85 (0.86)	n/a	0.62
			Cooperation with diabetes team	4 (3,7^r^,14^r^,19)	n/a	8.1 ±2.0	n/a	0.73 (0.74)	n/a	0.50

Displayed are mean ( ± SD) scale scores, Cronbach’s α and retest correlations for each DSMQ-R scale stratified by study and diabetes type.

CES-D, Center for Epidemiologic Studies Depression Scale; DM, diabetes mellitus; HbA_1c_, glycated hemoglobin; MDI, multiple daily (insulin) injections; n/a, not available; PAID, Problem Areas in Diabetes Scale; PHQ-9, Patient Health Questionnaire-9; T1D, type 1 diabetes; T2D, type 2 diabetes.

^1^ The 27-item total score, including the optional items 21–27 regarding intensive insulin treatment, was calculated for corresponding subgroups only (=full T1D group and those with T2D using bolus insulin and multiple daily insulin injections (MDI)).

^r^Reverse-scored item.

### Internal Reliability

Cronbach’s *α* of the 20-item total scale varied from 0.88–0.92 (mean=0.90) in T1D and from 0.84–0.89 (mean=0.87) in T2D across studies. Coefficients were slightly higher for the 27-item total scale, where applicable (**Table 2**). For the subscales, mean coefficients *α* for T1D (T2D) were: ‘eating behavior’=0.76 (0.78), ‘medication taking’=0.79 (0.75), ‘glucose monitoring’=0.76 (0.82), ‘physical activity’=0.87 (0.80) and ‘cooperation with diabetes team’=0.82 (0.67). McDonald’s *ω* yielded consistent results (**Table 2**). Direct comparisons of scale reliabilities estimated including the newly added items versus original ones only yielded consistently higher reliability coefficients for the new scales (**Supplementary Table 5**).

### Reproducibility

Retest correlations over three to six months reflected sufficient intra-individual stability of the measurement over time. Mean correlations for 20-item total scale were 0.64 in T1D and 0.53 in T2D; mean correlations for the subscales were from 0.53–0.69 in T1D and from 0.43–0.61 in T2D (**Table 2**).

### Construct Validity

Correlations with convergent criteria were generally in line with expectations towards validity of the measurement and a meaningful nomological network.

*Total scale:* Higher DSMQ-R total scores (suggesting more optimal self-management) were consistently associated with better HbA_1c_ values across studies and diabetes types; however, the sizes of associations varied (e.g., from -0.29 to -0.57, mean = -0.41, in T1D and from -0.20 to -0.36, mean=-0.30, in T2D; 20-item total). Higher DSMQ-R total scores were also associated with lower mean sensor glucose, more time in range, less time above range and lower glucose variability in T1D (study 4). Further, higher DSMQ-R total scores were associated with lower rates of long-term complications and less events of ketoacidosis (T1D). DSMQ-R total scores were highly positively associated with convergent measures of treatment motivation, treatment satisfaction and self-management performance according to the SDSCA questionnaire and corresponding DDS/T1-DDS items (**Table 3**); and highly negatively with items reflecting suboptimal treatment behavior. In studies 4 and 5, significant correlations with EMA items reflecting self-management were observed. Finally, higher DSMQ total scores were seen in people with better mental health, lower diabetes distress and less depressive symptoms.

**Table 3:** Validity and responsiveness indices for the DSMQ-R scales by study and diabetes type.

Studies	DSMQ scales	Convergent criteria/outcome variables	Correlations (Pearson’s *r*) with criteria/outcomes		Baseline to follow-up changes (Cohen’s *d*) per scale	
			T1D	T2D	T1D	T2D
Study 1: Cross-sectional questionnaire study (2015–16); *n*=588 PWD (333 with T1D, 255 with T2D)	20-item total score	HbA_1c_	-0.57‡	-0.36‡	n/a	n/a
		Ketoacidosis past year (yes)	-0.22‡	n/a		
		With complications^2^ (yes)	-0.17†	-0.20†		
		Treatment satisfaction (DTSQ score)	0.63‡	0.53‡		
		Treatment motivation (DAS subscale)	0.68‡	0.56‡		
		Treatment neglect (DAS subscale)	-0.77‡	-0.68‡		
		Diabetes distress (PAID-5 score)	-0.42‡	-0.27‡		
		Depressive symptoms (PHQ-9 score)	-0.49‡	-0.43‡		
	27-item total score^1^	HbA_1c_	-0.57‡	-0.49‡	n/a	n/a
		Ketoacidosis past year (yes)	-0.21†	n/a		
		With complications^2^ (yes)	-0.17†	-0.25*		
		Treatment satisfaction (DTSQ score)	0.64‡	0.53‡		
		Treatment motivation (DAS subscale)	0.71‡	0.61‡		
		Treatment neglect (DAS subscale)	-0.76‡	-0.69‡		
		Diabetes distress (PAID-5 score)	-0.47‡	-0.40‡		
		Depressive symptoms (PHQ-9 score)	-0.53‡	-0.48‡		
	Eating behavior	HbA_1c_	-0.43‡	-0.31‡	n/a	n/a
	Medication taking	HbA_1c_	-0.55‡	-0.42‡	n/a	n/a
	Glucose monitoring	HbA_1c_	-0.53‡	-0.15*	n/a	n/a
	Physical activity	BMI	-0.10	-0.28‡	n/a	n/a
	Cooperation with diabetes team	Self-reported *n* of diabetologist visits past year	0.14*	0.09	n/a	n/a
Study 2: ‘Depression and Diabetes Control Trial’ (2016–17), prospective randomized trial, retest after six months *n*=198 PWD (131 with T1D, 67 with T2D)	20-item total score	HbA_1c_	-0.36‡	-0.30*	Total sample: 5.8 ±1.9 to 6.8 ±1.5‡ (*d*=0.66). EG: 6.0 ±1.8 to 6.8 ±1.6† (*d*=0.50), CG: 5.7 ±1.9 to 6.8 ±1.4‡ (*d*=0.82), *p* (time*group)=0.66	Total sample: 5.3 ±1.5 to 6.1 ±1.6† (*d*=0.51). EG: 5.2 ±1.3 to 5.9 ±1.5† (*d*=0.51), CG: 5.8 ±1.9 to 6.8 ±1.5‡ (*d*=0.53), *p* (time*group)=0.89
		Summary of diabetes self-care activities past week (SDSCA total score)	0.76‡	0.77‡		
		Often failing with diabetes routine (DDS item 8)	-0.69‡	-0.54‡		
		Diabetes distress (PAID score)	-0.28‡	-0.24*		
		Diabetes acceptance (DAS score)	0.51‡	0.40†		
		Depressive symptoms (PHQ-9 score)	-0.28†	-0.22		
	27-item total score^1^	HbA_1c_	-0.39‡	-0.32*	Total sample: 5.8 ±1.9 to 6.9 ±1.4‡ (*d*=0.72). EG: 6.0 ±1.8 to 6.9 ±1.5‡ (*d*=0.59), CG: 5.6 ±2.0 to 6.9 ±1.4‡ (*d*=0.85), *p* (time*group)=0.49	Total sample^1^: 5.6 ±1.6 to 6.4 ±1.6† (*d*=0.57). EG: 5.3 ±1.1 to 6.5 ±1.2‡ (*d*=1.44), CG: 6.1 ±2.1 to 6.2 ±2.1 (*d*=0.06), *p* (time*group)=0.15
		Summary of diabetes self-care activities past week (SDSCA total score)	0.73‡	0.76‡		
		Often failing with diabetes routine (DDS item 8)	-0.70‡	-0.49‡		
		Diabetes distress (PAID score)	-0.31‡	-0.05		
		Diabetes acceptance (DAS score)	0.51‡	0.33*		
		Depressive symptoms (PHQ-9 score)	-0.31‡	-0.13		
	Eating behavior	HbA_1c_	-0.34‡	-0.01	Total sample: 5.0 ±2.2 to 5.7 ±1.9† (*d*=0.37). EG: 5.2 ±2.3 to 5.8 ±2.1 (*d*=0.30), CG: 4.9 ±2.2 to 5.7 ±1.8* (*d*=0.42), *p* (time*group)=0.93	Total sample: 3.9 ±1.8 to 5.0 ±1.8† (*d*=0.59). EG: 3.7 ±1.8 to 4.6 ±1.8† (*d*=0.51), CG: 4.3 ±2.0 to 5.9 ±1.8* (*d*=0.71), *p* (time*group)=0.68
		Healthy eating past week (SDSCA General Diet scale)	0.67‡	0.65‡		
		Not sticking to a good meal plan (DDS item 12)	-0.57‡	0.54‡		
	Medication taking	HbA_1c_	-0.43‡	-0.36†	Total sample: 7.8 ±2.7 to 8.8 ±1.8‡ (*d*=0.47). EG: 7.9 ±2.6 to 8.8 ±1.8* (*d*=0.43), CG: 7.7 ±2.9 to 8.8 ±1.9† (*d*=0.49), *p* (time*group)=0.74	Total sample: 8.0 ±2.5 to 8.6 ±2.1‡ (*d*=0.27). EG: 7.9 ±2.5 to 8.4 ±2.2 (*d*=0.23), CG: 8.3 ±2.5 to 9.0 ±1.9 (*d*=0.28), *p* (time*group)=0.51
		Self-reported *n* of daily insulin injections	0.27†	-0.14		
	Glucose monitoring	HbA_1c_	-0.29†	-0.39†	Total sample: 5.5 ±3.4 to 7.3 ±2.5‡ (*d*=0.61). EG: 5.6 ±3.3 to 7.0 ±2.7† (*d*=0.48), CG: 5.3 ±3.5 to 7.5 ±2.4‡ (*d*=0.73), *p* (time*group)=0.20	Total sample: 5.3 ±3.6 to 6.9 ±3.0‡ (*d*=0.64). EG: 4.7 ±3.6 to 6.6 ±3.0‡ (*d*=0.78), CG: 6.8 ±3.5 to 7.8 ±3.1* (*d*=0.39), *p* (time*group)=0.90
		SMBG past week (SDSCA Blood Sugar Testing scale)	0.59‡	0.81‡		
		Not testing sugar frequently enough (DDS item 6)	-0.74‡	-0.69‡		
		Self-reported *n* of daily SMBG checks	0.36‡	0.61‡		
	Physical activity	BMI	-0.05	-0.35†	Total sample: 5.1 ±3.0 to 5.4 ±3.0 (*d*=0.13). EG: 5.2 ±2.7 to 5.5 ±2.8 (*d*=0.12), CG: 4.9 ±3.3 to 5.3 ±3.3 (*d*=0.19), *p* (time*group)=0.99	Total sample: 4.2 ±2.4 to 3.9 ±2.6 (*d*=0.10). EG: 4.2 ±2.3 to 4.2 ±2.7 (*d*=0.00), CG: 4.4 ±2.7 to 3.2 ±2.1 (*d*=0.37), *p* (time*group)=0.23
		Physical exercise past week (SDSCA Exercise scale)	0.73‡	0.72‡		
	Cooperation with diabetes team	Self-reported *n* of diabetologist visits past half year	0.20*	0.35†	Total sample: 7.9 ±2.3 to 8.5 ±1.7* (*d*=0.28). EG: 8.0 ±2.3 to 8.5 ±1.9 (*d*=0.22), CG: 7.8 ±2.4 to 8.4 ±1.5 (*d*=0.28), *p* (time*group)=0.89	Total sample: 8.1 ±2.1 to 8.1 ±2.1 (*d*=0.00). EG: 8.1 ±1.8 to 8.3 ±1.8 (*d*=0.11), CG: 8.1 ±2.7 to 7.7 ±2.7 (*d*=0.17), *p* (time*group)=0.35
		Not having a diabetes doctor (DDS item 15)	-0.30†	-0.57‡		
Study 3: Five-year FU of the DIAMOS-ECCE HOMO cohort (2017–18), cross-sectional study *n*=298 PWD (191 with T1D, 107 with T2D)	20-item total score	HbA_1c_	-0.41‡	-0.34‡	n/a	n/a
		Often failing with diabetes routine (DDS item 8)	-0.64‡	-0.72‡		
		Diabetes distress (PAID score)	-0.32‡	-0.34‡		
		Diabetes acceptance (DAS score)	0.65‡	0.61‡		
		Depressive symptoms (PHQ-9 score)	-0.24†	-0.21*		
	27-item total score^1^	HbA_1c_	-0.42‡	-0.24*	n/a	n/a
		Often failing with diabetes routine (DDS item 8)	-0.61‡	-0.67‡		
		Diabetes distress (PAID score)	-0.26‡	-0.29†		
		Diabetes acceptance (DAS score)	0.63‡	0.57‡		
		Depressive symptoms (PHQ-9 score)	-0.21†	-0.26*		
	Eating behavior	HbA_1c_	-0.29‡	-0.35‡	n/a	n/a
		Not sticking to a good meal plan (DDS item 12)	0.50‡	0.54‡		
	Medication taking	HbA_1c_	-0.36‡	-0.35‡	n/a	n/a
		Self-reported *n* of daily insulin injections	0.01	0.06		
	Glucose monitoring	HbA_1c_	-0.36‡	-0.23*	n/a	n/a
		Not testing sugar frequently enough (DDS item 6)	-0.67‡	-0.72‡		
		Self-reported *n* of daily glucose checks/scans/ readings	0.33‡	0.39‡		
	Physical activity	BMI	-0.26‡	-0.25*	n/a	n/a
	Cooperation with diabetes team	Not having a diabetes doctor (DDS item 15)	-0.31‡	-0.14	n/a	n/a
Study 4: ‘DIA-LINK1’ (2019–20), prospective observational study, retest after three months *n*=203 PWT1D	20-item total score	HbA_1c_	-0.29‡	n/a	5.8 ±1.7 to 6.4 ±1.5‡ (*d*=0.38)	n/a
		Not giving diabetes as much attention as one should (T1-DDS item 28)	-0.61‡	n/a		
		Diabetes distress (PAID score)	-0.27‡	n/a		
		Diabetes acceptance (DAS-10 score)	0.40‡	n/a		
		Depressive symptoms (CES-D score)	-0.32‡	n/a		
		Feeling guilty for neglecting diabetes treatment (mean of daily EMA ratings)	-0.42‡	n/a		
		Feeling overwhelmed by diabetes treatment (mean of daily EMA ratings)	-0.23†	n/a		
		Mean sensor glucose (4 weeks)	-0.30‡	n/a		
		Time-below-range (<70 mg/dl)	-0.06	n/a		
		Time-in-range (70–180 mg/dl)	0.31‡	n/a		
		Time-above-range (>180 mg/dl)	-0.27‡	n/a		
		Glucose variability (CV)	-0.27‡	n/a		
	27-item total score^1^	HbA_1c_	-0.33‡	n/a	6.1 ±1.7 to 6.6 ±1.4‡ (*d*=0.37)	n/a
		Not giving diabetes as much attention as one should (T1-DDS item 28)	-0.61‡	n/a		
		Diabetes distress (PAID score)	-0.29‡	n/a		
		Diabetes acceptance (DAS-10 score)	0.41‡	n/a		
		Depressive symptoms (CES-D score)	-0.33‡	n/a		
		Feeling guilty for neglecting diabetes treatment (mean of daily EMA ratings)	-0.44‡	n/a		
		Feeling overwhelmed by diabetes treatment (mean of daily EMA ratings)	-0.24†	n/a		
		Mean sensor glucose (4 weeks)	-0.33‡	n/a		
		Time-below-range (<70 mg/dl)	-0.04	n/a		
		Time-in-range (70–180 mg/dl)	0.34‡	n/a		
		Time-above-range (>180 mg/dl)	-0.30‡	n/a		
		Glucose variability (CV)	-0.28‡	n/a		
	Eating behavior	HbA_1c_	-0.08	n/a	4.6 ±2.2 to 5.2 ±1.9‡ (*d*=0.35)	n/a
		Not eating as carefully as one should (T1-DDS item 2)	-0.55‡	n/a		
		Feeling that eating is out of control (T1-DDS item 23)	-0.48‡	n/a		
	Medication taking	HbA_1c_	-0.36‡	n/a	7.6 ±2.5 to 8.2 ±2.4† (*d*=0.25)	n/a
		Time-in-range (70–180 mg/dl)	0.33‡	n/a		
		Not taking as much insulin as one should (T1-DDS item 8)	-0.45‡	n/a		
		Self-reported *n* of daily insulin injections	0.22†	n/a		
	Glucose monitoring	HbA_1c_	-0.42‡	n/a	6.1 ±2.7 to 6.8 ±2.3‡ (*d*=0.29)	n/a
		Time-in-range (70–180 mg/dl)	0.36‡	n/a		
		Not checking glucose level as often as one should (T1-DDS item 12)	-0.70‡	n/a		
		Self-reported *n* of daily glucose checks/scans/ readings	0.40‡	n/a		
	Physical activity	BMI	-0.15*	n/a	5.5 ±3.2 to 5.6 ±3.0 (*d*=0.04)	n/a
	Cooperation with diabetes team	Can’t tell diabetes doctor what is on mind (T1-DDS item 7)	-0.27‡	n/a	8.1 ±2.2 to 8.3 ±1.9 (*d*=0.11)	n/a
		Diabetes doctor doesn’t understand what it’s like to have diabetes (T1-DDS item 18)	-0.20†	n/a		
Study 5: ‘DIA-LINK2’ (2020–21), prospective observational study, retest after three months *n*=180 PWT2D	20-item total score	HbA_1c_	n/a	-0.20†	n/a	5.4 ±1.9 to 6.4 ±1.8‡ (*d*=0.54)
		Often failing with diabetes routine (DDS item 8)	n/a	-0.40‡		
		Diabetes distress (PAID score)	n/a	-0.33‡		
		Diabetes acceptance (DAS-10 score)	n/a	0.46‡		
		Depressive symptoms (CES-D score)	n/a	-0.23†		
		Feeling guilty for neglecting diabetes treatment (mean of daily EMA ratings)	n/a	-0.43‡		
		Self-rated quality of self-management overall (mean of daily EMA ratings)	n/a	0.47‡		
	27-item total score^1^	HbA_1c_	n/a	-0.25†	n/a	5.8 ±1.9 to 6.9 ±1.7‡ (*d*=0.61)
		Often failing with diabetes routine (DDS item 8)	n/a	-0.43‡		
		Diabetes distress (PAID score)	n/a	-0.39‡		
		Diabetes acceptance (DAS-10 score)	n/a	0.54‡		
		Depressive symptoms (CES-D score)	n/a	-0.37‡		
		Feeling guilty for neglecting diabetes self- management (mean of daily EMA ratings)	n/a	-0.37‡		
		Self-rated quality of self-management overall (mean of daily EMA ratings)	n/a	0.44‡		
	Eating behavior	HbA_1c_	n/a	-0.08	n/a	4.2 ±2.3 to 5.6 ±2.3‡ (*d*=0.61)
		Not sticking to a good meal plan (DDS item 12)	n/a	-0.53‡		
		Diet promoting good glucose control (EMA ratings)	n/a	0.44‡		
		Diet beneficial for weight (EMA ratings)	n/a	0.46‡		
	Medication taking	HbA_1c_	n/a	-0.22†	n/a	7.4 ±3.0 to 8.1 ±2.3† (*d*=0.26)
		Self-reported *n* of daily insulin injections	n/a	0.00		
		Medical diabetes treatment appraisal (EMA ratings)	n/a	0.33‡		
	Glucose monitoring	HbA_1c_	n/a	-0.08	n/a	5.3 ±3.2 to 6.8 ±2.8‡ (*d*=0.50)
		Not testing sugar frequently enough (DDS item 6)	n/a	-0.57‡		
		Self-reported *n* of daily glucose checks/scans/readings	n/a	0.41‡		
	Physical activity	Level of physical activity contributing to good diabetes management (EMA ratings)	n/a	0.58‡	n/a	4.1 ±3.1 to 5.1 ±3.0‡ (*d*=0.36)
		BMI	n/a	-0.31‡		
		HbA_1c_	n/a	-0.15*		
	Cooperation with diabetes team	Not having a doctor for diabetes (DDS item 15)	n/a	-0.39‡	n/a	8.2 ±1.9 to 8.1 ±1.9 (*d*=0.05)

Displayed are criterion-related correlations and indices of change (Cohen’s d) from baseline to 6-month follow-up for each DSMQ-R scale stratified by study and diabetes type.

Indication of two-tailed significance: *p < 0.05, ^†^p < 0.01, ^‡^p < 0.001.

BMI, body mass index; CES-D, Center for Epidemiologic Studies Depression Scale; CG, control group; CV, coefficient of variation; DAS, Diabetes Acceptance Scale; DDS, Diabetes Distress Scale; DM, diabetes mellitus; DTSQ, Diabetes Treatment Satisfaction Questionnaire; EG, experimental group; EMA, ecological momentary assessment; HbA_1c_, glycated hemoglobin; iscCGM, intermittently scanned continuous glucose monitoring; MDI, multiple daily (insulin) injections; n/a, not available; PAID, Problem Areas in Diabetes Scale; PHQ-9, Patient Health Questionnaire-9; PWD, people with diabetes; rtCGM, real-time continuous glucose monitoring; SDSCA, Summary of Diabetes Self-Care Activities; SMBG, self-monitoring of blood glucose; T1-DDS, Type 1 Diabetes Distress Scale; T1D, type 1 diabetes; T2D, type 2 diabetes.

^1^The 27-item total score, including the optional items 21–27 regarding intensive insulin treatment, was calculated for corresponding subgroups only [=full T1D group and those with T2D using bolus insulin and multiple daily insulin injections (MDI)].

^2^Assessed were diabetic retinopathy, diabetic neuropathy, diabetic nephropathy, diabetic foot syndrome.

*Subscales:* The subscales ‘eating behavior’, ‘medication taking’ and ‘glucose monitoring’ showed significant associations with corresponding convergent criteria for diabetes-adjusted eating (e.g., SDSCA scale on healthy eating, DDS/T1-DDS items regarding sticking to a good meal plan and eating carefully), medication taking (e.g., T1-DDS item on insulin taking, mean number of daily insulin injections in T1D), glucose monitoring (e.g., SDSCA scale on blood sugar testing, DDS/T1-DDS items on glucose checking, mean number of daily glucose checks/scans). Each of the scales showed significant associations with better HbA_1c_ in several studies, however not all. The subscale ‘physical activity’ showed high correlations with the convergent SDSCA scale on past-week physical exercise and small-to-moderate associations with BMI. The subscale ‘cooperation with diabetes team’ showed significant correlations with self-reported frequencies of diabetologist visits as well as corresponding DDS/T1-DDS items on doctor-related problems. ‘Eating behavior’, ‘medication taking’ and ‘physical activity’ were also significantly associated with corresponding EMA ratings in studies 4 and 5.

### Structural Validity

Confirmatory factor analyses supported a five-factor structure representing the five subscales with excellent fit to the data for both T1D and T2D (**Supplementary Figures 1–2**). One-factor models representing the total scale showed good fit as well; however, with slightly lower fit indices and lower factor loadings (**Supplementary Figures 3–6**).

### Responsiveness

The ability to detect change was supported by significant changes over time in the total score and most subscale scores in the prospective studies. Greater changes were seen in the total scale and ‘eating behavior’ and ‘glucose monitoring’ subscales, while changes in ‘medication taking’ were modest and changes in ‘physical activity’ and ‘cooperation with diabetes team’ were small or lacking (**Table 3**). Between-group comparisons for people receiving depression treatment versus diabetes care as usual in study 2 suggested similar changes in DSMQ-R scores without significant differences between the groups at six-month follow-up.

## Discussion

### Main Findings

The evaluation of the DSMQ-R using data from diverse studies suggests very good properties in measuring diabetes self-management behavior in both T1D and T2D according to clinimetric criteria (40). Results suggest that the tool has good reliability, validity and responsiveness to change. The terms and expressions used in the questionnaire were updated to conform with modern diabetes-related language. The revised scales with newly added items showed higher internal reliability than the original version’s item sets.

The DSMQ-R total scale constitutes a reliable and valid measure of overall self-management. Yet it is a global measure; thus assessing the specific behaviors using the subscales may be preferred and even necessary for understanding individual aspects. For the subscales, however, differential properties and options should be considered: First, the numbers of items per scale differ which may affect reliability of the measurement. In this evaluation, most subscales yielded satisfactory to good reliability estimates; however, lower reliability coefficients were seen for subscales with fewer items (e.g., medication taking) in some of the studies. Furthermore, coefficients varied across studies and patient groups, suggesting that the utilization of subscales in research might benefit from affirming reliability within a given study data set. Notably, despite specific revisions and improved internal reliability, the ‘cooperation with diabetes team’ subscale still showed subthreshold reliability coefficients in two of five studies for T2D; yet not for T1D.

Reliability coefficients were mostly slightly higher in T1D subsamples compared to T2D which is in line with previous findings (21). This might be explained by more diverse treatment regimens and practices in T2D; for instance, prescribed medications may be diverse (oral drugs, insulin and/or incretin mimetics), glucose testing may or may not be required and dietary recommendations may vary in relevance and function. This might also explain higher associations between the DSMQ-R scales and HbA_1c_ in T1D [consistent with previous findings (23)], where glycemic outcomes directly depend on the consistent coordination and adjustment of carbohydrate intake, activities and insulin doses; whereas in T2D, glycemic control may rely more on diet and activity and less on glucose checking and meal-specific decisions (depending on the treatment regimen); also, residual insulin action may stabilize glycemic levels and reduce hyperglycemia.

It should be noted that two-sided questioning (using both positively and negatively keyed items) may lower internal consistency as observed in some DSMQ-R subscales; at the same time, higher validity is achieved and response bias is prevented. From a clinimetric perspective, a varied assessment using items covering different aspects from different sides is more important than a highly homogeneous measurement (40).

Validity of the scale measurement was supported by high correlations with convergent scores and items from other questionnaires. However, as self-report is prone to bias, associations with objective measures constitute another important source of information. Thus, the widely consistent associations between DSMQ-R scales and HbA_1c_ (as well as CGM-derived glucose parameters) across studies may be seen as extra evidence favoring validity.

Relatively good explanation of variation in HbA_1c_ was already observed in our previous studies for both T1D and T2D (21, 23). This might be explained by i) the reflection of behaviors over a broader, more representative reference period and ii) the items requesting behavioral evaluations (e.g., ‘with care and attention’) rather than behavior frequency (e.g., ‘on how many days…?’ as in the SDSCA). On the other hand, three studies using the DSMQ with non-Western samples (42–44) and one Hungarian study (45) have reported lower associations with HbA_1c_, suggesting caution against generalization across cultures.

Validity of the measurement was also supported by the structural representation of assessed contents (i.e., items and scales) in the factor analyses with good model fit for both T1D and T2D.

Change scores reflecting improvements in DSMQ-R-assessed behaviors supported good responsiveness of the measurement. In study 4, similar changes were seen for people randomized to depression treatment versus diabetes care as usual; this could be explained by both groups receiving treatment with beneficial effects on self-management behavior. The tool’s ability to detect change is also supported by findings from international studies using the DSMQ which found significant self-management improvement over time and between-group differences in randomized trials (46–49); notably, observed changes in DSMQ scores by group were often accompanied by parallel changes in HbA_1c_, which might be taken as evidence supporting validity of the changes (46, 48, 50). With regard to responsiveness and the tool’s reference period (eight weeks), a shorter period might facilitate the detection of short-term changes, thus adapting the instruction (e.g., four weeks), where needed, may be considered.

In terms of item amendments (e.g., revised wording), the DSMQ-R probably constitutes a relevant improvement. However, since most revisions were minor and item concepts were kept equivalent, the original 16-item version is basically included in the revised form. Estimation of scales as for the original version, where needed (e.g., to compare scores with former study results), would still be possible.

### Limitations and Strengths

The inferences drawn from this research are qualified by the following limitations: first, four of the studies whose data were analyzed here focused on diabetes-comorbid mental conditions, thus rates of depressive symptoms and/or diabetes distress were elevated and the samples may not be representative for the general population with diabetes (i.e., risk of spectrum bias). Second, we assessed cross-sectional associations between self-reported behavior and diabetes outcomes, thus inferences towards causation are not possible; in fact, associations with glycemic outcomes might be bidirectional; for instance, knowing of glycemic levels (e.g., last HbA_1c_) might influence self-management self-appraisal in the questionnaire. Third, the study samples were recruited within secondary or tertiary care, thus samples may not represent the primary care population; based on this, people with T2D assessed here used advanced medical treatments often including insulin and even basal-bolus therapy with multiple daily injections, whereas people with diet-and-exercise regimens and/or oral antidiabetic treatment alone were less represented.

The strengths of the evaluation may be seen in the standardized assessment using validated scales and items, temporal coincidence of questionnaire self-reports, interviews and laboratory measures and the inclusion of multiple methods including CGM and EMA for the assessment of convergent criteria. Furthermore, the stratified analyses for T1D and T2D using sufficiently large samples support evaluation for both major types of diabetes. Due to potential advantages of McDonald’s *ω* over Cronbach’s *α* (41), we calculated both estimates, yielding highly consistent results. Finally, the evaluation across different study samples, both general and specific, yields a more comprehensive and representative total evidence base; the fact that indices of reliability and validity, including associations with clinical criteria, were relatively consistent across studies may favor generalizability.

### Conclusions

In summary, the results support good clinimetric properties of the DSMQ-R. The tool can be used for research and clinical practice. It may help understand barriers and facilitators of functional self-management in T1D and T2D, facilitate the identification improvable practices in individuals and monitor behavior change following treatment in practical care or research trials.

## Data Availability Statement

The raw data supporting the conclusions of this article will be made available by the authors, without undue reservation.

## Ethics Statement

The studies involving human participants were reviewed and approved by Ethics Committee of the German Psychological Society or the Ethics Committee of the State Medical Chamber of Baden-Wuerttemberg. The patients/participants provided their written informed consent to participate in this study.

## Author Contributions

AS: developed and revised the DSMQ; planned and designed study 1; co-planned and designed studies 2–5; collected the data; performed the evaluation, analyzed and interpreted the data; wrote the manuscript. BK and NH: planned and designed studies 2–5; discussed the findings and revised the manuscript. DE: planned and designed studies 4–5; co-planned and designed studies 2–3; discussed the findings and revised the manuscript. TH: discussed the findings and revised the manuscript. All authors contributed to the article and approved the submitted version.

## Funding

Study 1 was supported by the German Diabetes Foundation (DDS) (grant number 375.10.15). Studies 2–3 were supported by the German Center for Diabetes Research (DZD) (grant number 82DZD01102). Studies 4–5 were supported by the German Center for Diabetes Research (DZD) [grant number 82DZD11A02]. The funders were not involved in decisions regarding study design; collection, analysis and interpretation of data; writing of the report; and submission of the article for publication.

## Conflict of Interest

The authors declare that the research was conducted in the absence of any commercial or financial relationships that could be construed as a potential conflict of interest.

## Publisher’s Note

All claims expressed in this article are solely those of the authors and do not necessarily represent those of their affiliated organizations, or those of the publisher, the editors and the reviewers. Any product that may be evaluated in this article, or claim that may be made by its manufacturer, is not guaranteed or endorsed by the publisher.

## Abbreviations

CES-D, Center for Epidemiologic Studies Depression Scale; CG, control group; CV, coefficient of variation; DAS, Diabetes Acceptance Scale; DDS, Diabetes Distress Scale; DM, diabetes mellitus; DSMQ, Diabetes Self-Management Questionnaire; DTSQ, Diabetes Treatment Satisfaction Questionnaire; EG, experimental group; EMA, ecological momentary assessment; HbA_1c_, glycated hemoglobin; iscCGM, intermittently scanned continuous glucose monitoring; MDI, multiple daily (insulin) injections; PAID, Problem Areas in Diabetes Scale; PHQ-9, Patient Health Questionnaire-9; PWD, people with diabetes; rtCGM, real-time continuous glucose monitoring; SDSCA, Summary of Diabetes Self-Care Activities; SMBG, self-monitoring of blood glucose; T1-DDS, Type 1 Diabetes Distress Scale; T1D, type 1 diabetes; T2D, type 2 diabetes.
