# Supplementary material for: A Self-Report Measure of Diabetes Self-Management for Type 1 and Type 2 Diabetes: The Diabetes Self-Management Questionnaire-Revised (DSMQ-R) – Clinimetric Evidence From Five Studies
Source: Front Clin Diabetes Healthc. 2022 Jan 13;2:823046. doi: 10.3389/fcdhc.2021.823046 (PMC10012087; doi:10.3389/fcdhc.2021.823046)
Supplement: Supplementary file 1 [file DataSheet_1.pdf]

frontiers

Supplementary Material

to *Schmitt A, Kulzer B, Ehrmann D, Haak T, Hermanns N. A self-report measure of diabetes self-management for type 1 and type 2 diabetes: the Diabetes Self-Management Questionnaire-Revised (DSMQ-R) – Clinimetric evidence from five studies. Front Clin Diabetes Healthc (2022).*

1. **Suppl. Table 1. Scoring information for the DSMQ-R**……………………………..………......pp. 2-4

2. **Suppl. Table 2. Available translations and languages of the DSMQ with published validation and other corresponding studies**………………………………………………….…..………..pp. 5-18

3. **Suppl. Table 3. Explanation and rationale of item revisions for the DSMQ-R** …………………….…………………………………………………………...……...…....pp. 19-21

4. **Suppl. Table 4. Ecological momentary assessment (EMA) items regarding diabetes self-management requested in the DIA-LINK studies.**…………….……………………......……......p. 22

5. **Suppl. Table 5. Internal reliability coefficients compared for scales including the new items versus original items only**……………………………..……………………………….....…pp. 23-25

6. **Suppl. Figure 1 / 2. Confirmatory factor analysis testing a five-factor model representing the DSMQ-R subscales on people with T1D / T2D**…………………………………………….……..pp.26-27

7. **Suppl. Figure 3 / 4. Confirmatory factor analysis testing a one-factor model representing the 20-item total scale on people with T1D / T2D**…………………………………...…….……..pp.28-29

8. **Suppl. Figure 5 / 6. Confirmatory factor analysis testing a one-factor model representing the 27-item total scale on people with T1D / T2D**……………………………………....……..pp.30-31

Suppl. Table 1. Scoring instructions for the DSMQ-R

Scoring for people with diabetes <u>without</u> intensive insulin treatment (using items 1 – 20)	
Total scale	· Includes all 20 items (items 16 and 20 are included in the total scale only).<br>· Negatively keyed = reverse-scored items: 5, 7, 10, 11, 12, 13, 14, 15, 16, 18, 20.<br>· Sum item scores after reverse-scoring all negatively keyed items.<br>· Transform to scale range 0–10: [sum score / 60 * 10]
‘Eating behavior’ subscale	· Includes six items: 2, 5, 9, 13, 17, 18.<br>· Negatively keyed = reverse-scored items: 5, 13, 18.<br>· Sum item scores (after reverse-scoring the negative items).<br>· Transform to scale range 0–10: [sum score / 18 * 10]
‘Medication taking’ subscale	· Includes two items: 4, 12.<br>· Negatively keyed = reverse-scored items: 12.<br>· Sum item scores (after reverse-scoring the negative item).<br>· Transform to scale range 0–10: [sum score / 6 * 10]
‘Glucose monitoring’ subscale	· Includes three items: 1, 6, 10.<br>· Negatively keyed = reverse-scored items: 10.<br>· Sum item scores (after reverse-scoring the negative items).<br>· Transform to scale range 0–10: [sum score / 9 * 10]
‘Physical activity’ subscale	· Includes three items: 8, 11, 15.<br>· Negatively keyed = reverse-scored items: 11, 15.<br>· Sum item scores (after reverse-scoring the negative items).<br>· Transform to scale range 0–10: [sum score / 9 * 10]
‘Cooperation with diabetes team’ subscale	· Includes four items: 3, 7, 14, 19.<br>· Negatively keyed = reverse-scored items: 7, 14.<br>· Sum item scores (after reverse-scoring the negative items).<br>· Transform to scale range 0–10: [sum score / 12 * 10]
Scoring for people with diabetes <u>with</u> intensive insulin treatment (using items 1 – 27)	
Total scale	· Includes all 20 items (items 16 and 20 are included in the total scale only). Items 21 to 27 can be added.<br>· Negatively keyed = reverse-scored items: 5, 7, 10, 11, 12, 13, 14, 15, 16, 18, 20.

	· Sum item scores after reverse-scoring all negatively keyed items.<br>· Transform to scale range 0–10:<br>[20-item sum score / 60 * 10]<br>[27-item sum score / 81 * 10]
‘Eating behavior’ subscale	· Includes six items: 2, 5, 9, 13, 17, 18.<br>· Negatively keyed = reverse-scored items: 5, 13, 18.<br>· Sum item scores (after reverse-scoring the negative items).<br>· Transform to scale range 0–10:<br>[sum score / 18 * 10]
‘Medication taking’ subscale	· Includes two items: 4, 12. Items 22, 23, 24 can be added.<br>· Negatively keyed = reverse-scored items: 12.<br>· Sum item scores (after reverse-scoring the negative item).<br>· Transform to scale range 0–10:<br>[2-item sum score / 6 * 10]<br>[5-item sum score / 15 * 10]
‘Glucose monitoring’ subscale	· Includes three items: 1, 6, 10. Item 21 can be added.<br>· Negatively keyed = reverse-scored items: 10.<br>· Sum item scores (after reverse-scoring the negative items).<br>· Transform to scale range 0–10:<br>[3-item sum score / 9 * 10]<br>[4-item sum score / 12 * 10]
‘Physical activity’ subscale	· Includes three items: 8, 11, 15.<br>· Negatively keyed = reverse-scored items: 11, 15.<br>· Sum item scores (after reverse-scoring the negative items).<br>· Transform to scale range 0–10:<br>[sum score / 9 * 10]
‘Cooperation with diabetes team’ subscale	· Includes four items: 3, 7, 14, 19.<br>· Negatively keyed = reverse-scored items: 7, 14.<br>· Sum item scores (after reverse-scoring the negative items).<br>· Transform to scale range 0–10:<br>[sum score / 12 * 10]
The additional items 21 to 27 might also be aggregated to an additional ‘adjusting/timing insulin doses and correcting high/low glucose levels’ subscale (after affirming sufficient scale consistency). However, this subscale will have high overlap with the ‘Medication taking’ and ‘Glucose monitoring’ subscales.	· Includes seven items: 21, 22, 23, 24, 25, 26, 27.<br>· Negatively keyed = reverse-scored items: none.<br>· Sum item scores.<br>· Transform to scale range 0–10:<br>· [sum score / 21 * 10]
General recommendations and considerations	
<u>*Re summing items to scales:*</u><br>· We recommend to check and approve sufficient internal consistency using the corresponding items before summing to scales.<br>· Estimate scale scores ranging from 0 to 10 using the following general formula:	

Actual sum of item scores divided by *maximum possible sum of items* (= item number * 3) multiplied with 10.

- If an item was skipped, the numerator (maximum possible sum of items) should be discounted by 3 points.
- If more than half of the items of a scale were skipped, adding up to a scale score should be avoided.

Re detecting suboptimal self-management practices/people in need of support:

- If categorization is required, establishing a cut-off score based on a given patient population (considering different person characteristics and treatment modalities) is recommended. A median or quartile split or score determination using distribution characteristics (e.g., one SD from the mean) or ROC analysis against an standard criterion should always be performed based on the given sample data rather than generalized from other study findings. Criteria are likely to differ across diabetes types, regimens and cultural backgrounds.
- If criteria for detecting people with suboptimal self-management practices are required, establishing an individual, conservative cut-off score would be preferred. With a view to response categories reflecting suboptimal or problematic behaviors (e.g., many items of a scale scored with 0 or 1 = low engagement in the assessed behavior), this might suggest a cut-off score at about 4 or 5 out of 10 for any scale.

  Referring to study 1 (*n*=333 T1D, *n*=255 T2D), a preliminary **cut-off score of ≤ 5** would suggest:

  - suboptimal eating behavior in 41% of PWT1D and 42% of PWT2D,
  - suboptimal medication taking in 19% of PWT1D and 13% of PWT2D,
  - suboptimal glucose monitoring in 32% of PWT1D and 27% of PWT2D,
  - suboptimal physical activity in 42% of PWT1D and 55% of PWT2D,
  - suboptimal cooperation with diabetes team in 13% of PWT1D and 16% of PWT2D.

  However, this is certainly an arbitrary criterion, and it is very difficult to find a general threshold for suboptimal behavior. Other criteria may be considered and given preference.

Suppl. Table 2. Available translations and languages of the DSMQ with published validations and other corresponding studies

Language	Translated/adapted by	Validation results published[1]	Studies using this or another translation in that language[1]
Albanian for Kosovo	Idriz Sopjani et al., Nursing Faculty, AAB College, Pristine, Kosovo	N/A	Sopjani I, Vehapi S, Gorani D, Imeri M, Vitoja S, Tahiri S. The Relation Between Depressive Symptoms and Self-Care in Patients with Diabetes Mellitus Type 2 in Kosovo. Med Arch (2016) 70:425-8. DOI: 10.5455/medarh.2016.70.425-428
Arabic for Algeria	Linguistic validation by Mapi Research Trust[2]	N/A	N/A
Arabic for Egypt	Ayman Abdelgalil M Elkady, Psychology Department, Tanta University, Egypt	Elkady AAM. Self-Care Management, Emotional Distress and Self-Efficacy: Relationships with Health-Related Quality of Life among Patients with Type 2 Diabetes. Int J Psycho-Educ Sci (2019) 8:73-84. Available from https://eric.ed.gov/?id=EJ1250728	Elkady AAM. Self-Care Management, Emotional Distress and Self-Efficacy: Relationships with Health-Related Quality of Life among Patients with Type 2 Diabetes. Int J Psycho-Educ Sci (2019) 8:73-84.
Arabic for Egypt	Linguistic validation by Mapi Research Trust[2]	N/A	N/A
Arabic for Jordan	Sawsan Hammad, Department of Community Health Nursing, University of Jordan, Amman, Jordan	Hammad S, Darawad M, Hourani E, Demeh W. Predictors of Glycated Hemoglobin among Jordanian Diabetic Patients. Iran J Public Health (2015) 44:1482-91. PMCID: PMC4703227	Darawad MW, Hammad S, Samarkandi OA, Hamdan-Mansour AM, Khalil AA. Evaluating the Psychometric Properties of the Arabic Version of the Diabetes Distress Scale. J Psychosoc Nurs Ment Health Serv (2017) 55:43-51. DOI: 10.3928/02793695-20170818-12
Arabic for Kuwait	Linguistic validation by Mapi Research Trust[2]	N/A	Al-Khaledi M, Al-Dousari H, Al-Dhufairi S, Al-Mousawi T, Al-Azemi R, Al-Azimi F, Badr HE. Diabetes Self-Management: A Key to Better Health-Related Quality of Life in Patients with Diabetes. Med Princ Pract (2018) 27:323-31. DOI: 10.1159/000489310
Arabic for Oman	Rajaa Al-Hadhrami, College of Nursing, Sultan Qaboos University, Sib, Oman<br>Linguistic validation by Mapi Research Trust[2]	N/A	Al-Hadhrami R, Al-Rawajfah O, Muliira J. Diabetes Self-Management and the Associated Factors Among Adult Omanis with Type 1 Diabetes. Sultan Qaboos Univ Med J (2020) 20:e339-45. DOI: 10.18295/squmj.2020.20.04.010

**Language**	**Translated/adapted by**	**Validation results published**[1]	**Studies using this or another translation in that language**[1]
Arabic for Saudi Arabia	Linguistic validation by Mapi Research Trust[2]	N/A	· Hassan SME. Adherence to Diabetic Self-Care Activities in Adolescent and Factors Contributing to Their Management within Sakaka City in Saudi Arabia. Int J Nurs Health Sci (2017) 4:37-45.<br>· Al shayban DM. Using Diabetes Self-Management Questionnaire (DSMQ) to Assess Diabetes Self-Care Activities for Diabetes Patients in King Fahad University Hospital - Saudi Arabia. Value in Health (2017) 20:PA482-A483. DOI: 10.1016/j.jval.2017.08.475<br>· Almigbal TH, Almutairi KM, Vinluan JM, Batais MA, Alodhayani A, Alonazi WB, Sheshah E, Alhoqail RI. Association of health literacy and self-management practices and psychological factor among patients with type 2 diabetes mellitus in Saudi Arabia. Saudi Med J (2019) 40: 1158-66. DOI: 10.15537/smj.2019.11.24585<br>· Al-Qahtani AM. Frequency and factors associated with inadequate self-care behaviors in patients with type 2 diabetes mellitus in Najran, Saudi Arabia. Based on diabetes self-management questionnaire. Saudi Med J (2020) 41:955-64. DOI: 10.15537/smj.2020.9.25339<br>· Alruhaim HY, Almigbal TH, Almutairi JS, Mujammami MH, AlMogbel TA, Alrasheed AA, Al Zahrani AM, Batais MA. The association between diabetes numeracy and diabetes self-management among Saudi adults with insulin-treated diabetes. Saudi Med J (2021) 42:517-25. DOI: 10.15537/smj.2021.42.5.20200422<br>· Alhussein NA, Mahzari MM, Aljumaie NM, Alosaimi MI, Almansouf AS, Alkahtani FK. Diabetes Self-Management Among Healthcare Providers in King Abdulaziz Medical City, Riyadh: A Cross-Sectional Pilot Study. Cureus (2021) 13:e18155. DOI: 10.7759/cureus.18155<br>· Alodhayani A, Almutairi KM, Vinluan JM, Almigbal TH, Alonazi WB, Ali Batais M, Mohammed Alnassar M. Association between self-care management practices and glycemic control of patients with type 2 diabetes mellitus in

Language	Translated/adapted by	Validation results published[1]	Studies using this or another translation in that language[1]
			Saud Arabia: A cross-sectional study. Saudi J Biol Sci (2021) 28:2460-65. DOI: 10.1016/j.sjbs.2021.01.047
Arabic for the United Arab Emirates	Linguistic validation by Mapi Research Trust[2]	N/A	N/A
Bahasa Melayu for Malaysia	Syahnaz Mohd Hashim, Department of Family Medicine, Universiti Kebangsaan Malaysia Medical Centre, Malaysia	N/A	N/A
Bengali for India	Translation and linguistic validation by Mapi Research Trust[2]	N/A	Chandra M, Raveendranathan D, Johnson Pradeep R, Patra S, Rushi, Prasad K, Brar JS. Managing Depression in Diabetes Mellitus: A Multicentric Randomized Controlled Trial Comparing Effectiveness of Fluoxetine and Mindfulness in Primary Care: Protocol for DIAbetes Mellitus ANd Depression (DIAMAND) Study. Indian J Psychol Med (2020) 42:S31-8. DOI: 10.1177/0253717620971200
Chinese for China	Li Chaoqun et al., Hebei University, Baoding, China	N/A	N/A
English for Australia	Andreas Schmitt, Research Institute of the Diabetes Academy Mergentheim, Bad Mergentheim, Germany	· Schmitt A, Gahr A, Hermanns N, Kulzer B, Huber J, Haak T. The Diabetes Self-Management Questionnaire (DSMQ): development and evaluation of an instrument to assess diabetes self-care activities associated with glycaemic control. Health Qual Life Outcomes (2013) 11:138. DOI: 10.1186/1477-7525-11-138.<br>· Schmitt A, Reimer A, Hermanns N, Huber J, Ehrmann D, Schall S, Kulzer B. Assessing Diabetes Self-Management with the Diabetes Self-Management Questionnaire (DSMQ) Can Help Analyse Behavioural Problems Related to	Maneze D, Everett B, Astorga C, Yogendran D, Salamonson Y. The Influence of Health Literacy and Depression on Diabetes Self-Management: A Cross-Sectional Study. J Diabetes Res (2016) 2016:3458969. DOI: 10.1155/2016/3458969

Language	Translated/adapted by	Validation results published[1]	Studies using this or another translation in that language[1]
		Reduced Glycaemic Control. PLoS One (2016) 11:e0150774. DOI: 10.1371/journal.pone.0150774<br>· Schmitt A, Kulzer B, Ehrmann D, Haak T, Hermanns N. A self-report measure of diabetes self-management for type 1 and type 2 diabetes: the Diabetes Self-Management Questionnaire-Revised (DSMQ-R) – Clinimetric evidence from five studies. Front Clin Diabetes Healthc (2022); in press.	
English for Ghana	N/A	N/A	Apini R, Annan R, Apprey C, Asamoah-Boakye O. Predictors of Glycaemic Control among Ghanaian Type 2 Diabetic Patients Using Diabetes Self-management Approach. IJBCRR (2018) 23:1-18. DOI: 10.9734/IJBCRR/2018/42976
English for Nigeria	N/A	Babatunde S, Onu R. Psychometric Performance of the Diabetes Self-Management Questionnaire (DSMQ) Among Individuals Attending a Referral Hospital in Port Harcourt, Nigeria. SM J Public Health Epidemiol (2018) 4:1047.	Ojewale LY, Oluwatosin AO, Fasanmade AA, Odusan O. A survey on patients' characteristics, perception of family support and diabetes self-management among type 2 diabetes patients in South-West Nigeria. Nurs Open (2019) 6:208-15. DOI: 10.1002/nop2.236
English for the UK	Andreas Schmitt, Research Institute of the Diabetes Academy Mergentheim, Bad Mergentheim, Germany	· Schmitt A, Gahr A, Hermanns N, Kulzer B, Huber J, Haak T. The Diabetes Self-Management Questionnaire (DSMQ): development and evaluation of an instrument to assess diabetes self-care activities associated with glycaemic control. Health Qual Life Outcomes (2013) 11:138. DOI: 10.1186/1477-7525-11-138.<br>· Schmitt A, Reimer A, Hermanns N, Huber J, Ehrmann D, Schall S, Kulzer B. Assessing Diabetes Self-Management with the Diabetes Self-Management Questionnaire (DSMQ) Can Help	Huber JW, Hood G, Schmitt A, Fang ML, Callender M. Introducing an effective predictor of HbA1c for research and clinical care – the DSMQ (Diabetes Self-Management Questionnaire). Poster session presented at Diabetes UK Professional Conference 2014, Liverpool, United Kingdom.

Language	Translated/adapted by	Validation results published[1]	Studies using this or another translation in that language[1]
		Analyse Behavioural Problems Related to Reduced Glycaemic Control. PLoS One (2016) 11:e0150774. DOI: 10.1371/journal.pone.0150774<br>· Schmitt A, Kulzer B, Ehrmann D, Haak T, Hermanns N. A self-report measure of diabetes self-management for type 1 and type 2 diabetes: the Diabetes Self-Management Questionnaire-Revised (DSMQ-R) – Clinimetric evidence from five studies. Front Clin Diabetes Healthc (2022); in press.	
English for the US	Andreas Schmitt, Research Institute of the Diabetes Academy Mergentheim, Bad Mergentheim, Germany	· Schmitt A, Gahr A, Hermanns N, Kulzer B, Huber J, Haak T. The Diabetes Self-Management Questionnaire (DSMQ): development and evaluation of an instrument to assess diabetes self-care activities associated with glycaemic control. Health Qual Life Outcomes (2013) 11:138. DOI: 10.1186/1477-7525-11-138.<br>· Schmitt A, Reimer A, Hermanns N, Huber J, Ehrmann D, Schall S, Kulzer B. Assessing Diabetes Self-Management with the Diabetes Self-Management Questionnaire (DSMQ) Can Help Analyse Behavioural Problems Related to Reduced Glycaemic Control. PLoS One (2016) 11:e0150774. DOI: 10.1371/journal.pone.0150774<br>· Schmitt A, Kulzer B, Ehrmann D, Haak T, Hermanns N. A self-report measure of diabetes self-management for type 1 and type 2 diabetes: the Diabetes Self-Management Questionnaire-Revised (DSMQ-R) – Clinimetric evidence from	· Altshuler L, Plaksin J, Zabar S, Wallach A, Sawicki C, Kundrod S, Kalet A. Transforming the Patient Role to Achieve Better Outcomes Through a Patient Empowerment Program: A Randomized Wait-List Control Trial Protocol. JMIR Res Protoc (2016) 5:e68. DOI: 10.2196/resprot.5376<br>· Fearon-Lynch JA, Sethares KA, Asselin ME, Batty K, Stover CM. Effects of Guided Reflection on Diabetes Self-Care: A Randomized Controlled Trial. Diabetes Educ (2019) 45:66-79. DOI: 10.1177/0145721718816632<br>· Jia J, Quintiliani LM, Truong V, Jean C, Branch J, Lasser KE. A community-based diabetes group pilot incorporating a community health worker and photovoice methodology in an urban primary care practice. Cogent Medicine (2019) 6:1567973. DOI: 10.1080/2331205X.2019.1567973<br>· Khairnar R, Kamal KM, Giannetti V, Dwibedi N, McConaha J. Barriers and facilitators to diabetes self-management in a primary care setting - Patient perspectives. Res Social Adm Pharm (2019) 15:279-86. DOI: 10.1016/j.sapharm.2018.05.003<br>· Navarro D, Alpert P, Cross C. The Impact of Shift Work on Diabetes Self-Management Activities. J Dr Nurs Pract (2019) 12:66-72. DOI: 10.1891/2380-9418.12.1.66<br>· Turrin KB, Trujillo JM. Effects of Diabetes Numeracy on Glycemic Control and Diabetes Self-Management

Language	Translated/adapted by	Validation results published[1]	Studies using this or another translation in that language[1]
		five studies. Front Clin Diabetes Healthc (2022); in press.	Behaviors in Patients on Insulin Pump Therapy. Diabetes Ther (2019) 10:1337-46. DOI: 10.1007/s13300-019-0634-2<br>· Wade T, Fahey O, Vanmieghem M, Vivian E. Self-care behaviors of food insecure persons with diabetes. Health Edu Care (2019) 4:1-4. DOI: 10.15761/HEC.1000154<br>· Jia J, Jenkins AJ, Quintiliani LM, Truong V, Lasser KE. Resilience and diabetes self-management among African-American men receiving primary care at an urban safety-net hospital: a cross-sectional survey. Ethn Health (2020) 29:1-10. DOI: 10.1080/13557858.2020.1849566<br>· Summers-Gibson L. The Relationships Between Diabetes Self-Care, Diabetes Time Management, and Diabetes Distress in Women With Type 2 Diabetes Mellitus. Sci Diabetes Self Manag Care (2021) 47:245-54. DOI: 10.1177/26350106211014438<br>· Chaytor NS, Barbosa-Leiker C, Germine LT, Fonseca LM, McPherson SM, Tuttle KR. Construct validity, ecological validity and acceptance of self-administered online neuropsychological assessment in adults. Clin Neuropsychol (2021) 35:148-64. DOI: 10.1080/13854046.2020.1811893
French for Algeria	Linguistic validation by Mapi Research Trust[1]	N/A	N/A
German for Germany	Andreas Schmitt, Research Institute of the Diabetes Academy Mergentheim, Bad Mergentheim, Germany	· Schmitt A, Gahr A, Hermanns N, Kulzer B, Huber J, Haak T. The Diabetes Self-Management Questionnaire (DSMQ): development and evaluation of an instrument to assess diabetes self-care activities associated with glycaemic control. Health Qual Life Outcomes (2013) 11:138. DOI: 10.1186/1477-7525-11-138.<br>· Schmitt A, Reimer A, Hermanns N, Huber J, Ehrmann D, Schall S, Kulzer B. Assessing Diabetes Self-Management	· Nobis S, Lehr D, Ebert DD, Baumeister H, Snoek F, Riper H, Berking M. Efficacy of a web-based intervention with mobile phone support in treating depressive symptoms in adults with type 1 and type 2 diabetes: a randomized controlled trial. Diabetes Care (2015) 38:776-83. DOI: 10.2337/dc14-1728<br>· Brenk-Franz K, Strauss B, Tiesler F, Fleischhauer C, Ciechanowski P, Schneider N, Gensichen J. The Influence of Adult Attachment on Patient Self-Management in Primary Care--The Need for a Personalized Approach and Patient-Centred Care. PLoS One (2015) 10:e0136723. DOI: 10.1371/journal.pone.0136723

Language	Translated/adapted by	Validation results published[1]	Studies using this or another translation in that language[1]
		with the Diabetes Self-Management Questionnaire (DSMQ) Can Help Analyse Behavioural Problems Related to Reduced Glycaemic Control. PLoS One (2016) 11:e0150774. DOI: 10.1371/journal.pone.0150774<br>· Schmitt A, Kulzer B, Ehrmann D, Haak T, Hermanns N. A self-report measure of diabetes self-management for type 1 and type 2 diabetes: the Diabetes Self-Management Questionnaire-Revised (DSMQ-R) – Clinimetric evidence from five studies. Front Clin Diabetes Healthc (2022); in press.	· Ebert DD, Nobis S, Lehr D, Baumeister H, Riper H, Auerbach RP, Snoek F, Cuijpers P, Berking M. The 6-month effectiveness of Internet-based guided self-help for depression in adults with Type 1 and 2 diabetes mellitus. Diabet Med (2017) 34:99-107. DOI: 10.1111/dme.13173<br>· Schmitt A, Reimer A, Hermanns N, Kulzer B, Ehrmann D, Krichbaum M, Huber J. Haak T. Depression is linked to hyperglycaemia via suboptimal diabetes self-management: A cross-sectional mediation analysis. J Psychosom Res 2017;94:17-23. DOI: 10.1016/j.jpsychores.2016.12.015<br>· Schnell O, Klausmann G, Gutschek B, Garcia-Verdugo RM, Hummel M. Impact on Diabetes Self-Management and Glycemic Control of a New Color-Based SMBG Meter. J Diabetes Sci Technol (2017) 11:1218-25. DOI: 10.1177/1932296817706376<br>· Schmitt A, Reimer A, Kulzer B, Icks A, Paust R, Roelver KM, Kaltheuner A, Ehrmann D, Krichbaum M, Haak T, Hermanns N. Measurement of psychological adjustment to diabetes with the Diabetes Acceptance Scale. J Diabetes Complications (2018) 32:384-92. DOI: 10.1016/j.jdiacomp.2018.01.005<br>· Schmitt A, Bendig E, Baumeister H, Hermanns N, Kulzer B. Associations of Depression and Diabetes Distress with Self-Management Behaviour and Glycaemic Control in Type 1 and Type 2 Diabetes. Health Psychol (2021) 40:113-24. DOI: 10.1037/hea0001037
Gujarati for India	Translation and linguistic validation by Mapi Research Trust[2]	N/A	N/A
Hindi for India	Translation and linguistic validation by Mapi Research Trust[2]	N/A	· Adav S. Mental Health Problems and Self-Management Among People With Type-2 Diabetes. Indian J Health Wellbeing (2016) 7:1021-23.<br>· Khan MS, Mahmood SE, Ahmad A, Khan AA. Assessment of Self-Care Activities Using Diabetes Self-Management Questionnaire (DSMQ) amongst Diabetes Patients Attending a Rural Health Training Centre in Lucknow. J

Language	Translated/adapted by	Validation results published[1]	Studies using this or another translation in that language[1]
			Evolution Med Dent Sci (2021) 10:1324-8. DOI: 10.14260/jemds/2021/279<br>· Mahmood ZS, ElMoosaui AS, Abbas BF, Kamil MY, Muhammad ANH. Correlation between Glycemic Control and Diabetes Self-Care in Patients with Type 1 Diabetes on Insulin Regimens (Basalbolus vs. Biphasic premixed). Indian J Public Health Res Dev (2019) 10:2482-7. DOI: 10.5958/0976-5506.2019.02239.3
Hungarian	Agnes Vincze et al., Department of Internal Medicine and Oncology, Semmelweis University, Budapest, Hungary	Vincze A, Losonczi A, Stauder A. The validity of the diabetes self-management questionnaire (DSMQ) in Hungarian patients with type 2 diabetes. Health Qual Life Outcomes (2020) 18:344. DOI: 10.1186/s12955-020-01595-7	N/A
Indonesian for Indonesia	Lathifani Azka, Universitas Padjadjaran, Fakultas Keperawatan, Bandung, Indonesia	N/A	· Pamungkas RA, Chamroonsawasdi K. Self-management based coaching program to improve diabetes mellitus self-management practice and metabolic markers among uncontrolled type 2 diabetes mellitus in Indonesia: A quasi-experimental study. Diabetes Metab Syndr (2020) 14:53-61. DOI: 10.1016/j.dsx.2019.12.002<br>· Samudera WS, Efendi F, Indarwati R. Effect of community and peer support based healthy lifestyle program (CP-HELP) on self care behavior and fasting blood glucose in patient with type 2 Diabetes Mellitus. J Diabetes Metab Disord (2021) 20:193-99. DOI: 10.1007/s40200-021-00729-y<br>· Wuri Kartika A, Widyatuti W, Rekawati E. The effectiveness of home-based nursing intervention in the elderly with recurrent diabetic foot ulcers: A case report. J Public Health Res (2021) 10:2162. DOI: 10.4081/jphr.2021.2162
Italian for Italy	Unknown	N/A	· Balduzzi A, Marchegiani G, Andrianello S, Romeo F, Amodio A, De Pretis N, Zamboni G, Malleo G, Frulloni L, Salvia R, Bassi C. Pancreaticoduodenectomy for paraduodenal pancreatitis is associated with a higher

Language	Translated/adapted by	Validation results published[1]	Studies using this or another translation in that language[1]
			incidence of diabetes but a similar quality of life and pain control when compared to medical treatment. Pancreatology (2020) 20:193-8. DOI: 10.1016/j.pan.2019.12.014
Kannada for India	Translation and linguistic validation by Mapi Research Trust[2]<br>Prabhath M Kalkura, Manipal University, Manipal, India	N/A	Karthickeyan K, Panneerselvam P, Abhilash T, Kameswaran R, Krishnaveni K, Srinivasan S. Assessment of Therapeutic Outcome and Medication Adherence in Diabetics Consumed Insulin, Oral Hypoglycemics and Poly Herbal Drugs. J Young Pharm (2018) 10:226-30. DOI: 10.5530/jyp.2018.10.50
Lithuanian for Lithuania	Greta Raudonyte et al., Department of Health Psychology at the Vilnius University, Lithuania	N/A	N/A
Macedonian for Macedonia	Biljana Indova, Faculty of Pharmacy, University St. Cyril and Methodius, Skopje, Macedonia	N/A	N/A
Malay for Malaysia	Ju Ying Ang, Hospital Raja Permaisuri Bainun, Ipoh, Malaysia	N/A	N/A
Malayalam for India	Translation and linguistic validation by Mapi Research Trust[2]	N/A	N/A
Mandarin for China	Ju Ying Ang, Hospital Raja Permaisuri Bainun, Ipoh, Malaysia<br>Linguistic validation by Mapi Research Trust[2]	N/A	N/A
Mandarin for Malaysia	Linguistic validation by Mapi Research Trust[2]	N/A	N/A
Mandarin for Singapore	Janice Quek et al., James Cook University, Singapore	N/A	Quek J, Tan G, Lim K, Yap CK, Wong M, Soon J. Diabetes distress and self-management in primary care in Singapore: explorations through illness perception. Int J Community Med

**Language**	**Translated/adapted by**	**Validation results published**[1]	**Studies using this or another translation in that language**[1]
			Public Health (2019) 6:473-9. DOI: 10.18203/2394-6040.ijcmph20190166
Marathi for India	Translation and linguistic validation by Mapi Research Trust[2]	N/A	Kakade AA, Mohanty IR, Rai S. Assessment of knowledge, attitude and self-care activities among type-2 diabetic patients attending a tertiary care teaching hospital. Int J Basic Clin Pharmacol (2016) 5:2458-62. DOI: 10.18203/2319-2003.ijbcp20164105
Oriya for India	Translation and linguistic validation by Mapi Research Trust[2]	N/A	N/A
Persian for Iran	Mehran Nakhaeizadeh, Department of Biostatistics and Epidemiology, Faculty of Health, Kerman University of Medical Sciences, Kerman, Iran, & Ali Khaloei, Social Determinants of Health Research Center, Institute for Futures Studies in Health, Kerman University of Medical Sciences, Kerman, Iran	Nakhaeizadeh M, Khalooei A. Psychometric properties of the persian version of the diabetes self-management questionnaire for patients with Type 2 diabetes in Iran. Int J Prev Med (2021) 12:120. DOI: 10.4103/ijpvm.IJPVM_241_19	· Mehravar F, Mansournia MA, Holakouie-Naieni K, Nasli-Esfahani E, Mansournia N, Almasi-Hashiani A. Associations between diabetes self-management and microvascular complications in patients with type 2 diabetes. Epidemiol Health (2016) 38:e2016004. DOI: 10.4178/epih.e2016004<br>· Namjoo Nasab M, Ghavam A, Yazdanpanah A, Jahangir F, Shokrpour N. Effects of Self-management Education Through Telephone Follow-up in Diabetic Patients. Health Care Manag (Frederick) (2017) 36:273-81. DOI: 10.1097/HCM.0000000000000172<br>· Azami G, Soh KL, Sazlina S-G, Salmiah MS, Khosravi A, Aazami S, Valizadeh R. The Effect of Depression on Poor Glycemic Control in Adults with Type 2 Diabetes: The Mediating Roles of Self-Efficacy and Self-Management Behaviors. Int J Diabetes Metab (2019) 24:3-4. DOI: 10.1159/000502126<br>· Asghari F, Nobahar M. Comparison of self-care in non-cardiac diabetic patients. Diabetes Metab Syndr Obes (2019) 12:1675-83. DOI: 10.2147/DMSO.S209651<br>· Khalooei A, Benrazavy L. Diabetes Self-management and Its Related Factors among Type 2 Diabetes Patients in Primary Health Care Settings of Kerman, Southeast Iran. J Pharm Res Int (2019) 29:1-9. DOI: 10.9734/jpri/2019/v29i430241
	Fariba Hosseinzadegan et al., Urmia University of Medical Sciences, Urmia, Iran	Hosseinzadegan F, Azimzadeh R, Parizad N, Esmaeili R, Alinejad V, Maslakpak MH. Psychometric evaluation of the Diabetes Self-Management Questionnaire-Revised Form (DSMQ-R) in patients with diabetes. Nurs Midwifery J (2021) 19:109-18. http://unmf.umsu.ac.ir/article-1-4190-en.html	

<table>
<tr><th>Language</th><th>Translated/adapted by</th><th>Validation results published[1]</th><th>Studies using this or another translation in that language[1]</th></tr>
<tr><td></td><td></td><td></td><td>· Kalhor N, Afshari S, Vahedian M, Shajari R, Sharifimoghadam S, Tabarraii R. Evaluation of the relationship between different factors of self-management and control of diabetes in diabetic patients group. Journal of Preventive Epidemiology (2021) 6:18. DOI: 10.34172/jpe.2021.18</td></tr>
<tr><td rowspan="2">Portuguese for Brazil</td><td>Marilia Estevam Cornénio and Talita Maciel, School of Nursing, University of Campinas, Campinas, Brazil</td><td rowspan="2">Maciel T. Autocuidado no diabetes: adaptação cultural e avaliação das propriedades da medida do ?Diabetes Self-Management Questionnaire? (DSMQ); 2019. Dissertação. (unpublished dissertation, 2019)</td><td rowspan="2">N/A</td></tr>
<tr><td>Linguistic validation by Mapi Research Trust[2]</td></tr>
<tr><td>Portuguese for Portugal</td><td>Maria Alice dos Santos Curado, Superior Nursing School of Lisbon, Lisbon, Portugal</td><td>N/A</td><td>N/A</td></tr>
<tr><td>Punjabi for India</td><td>Translation and linguistic validation by Mapi Research Trust[2]</td><td>N/A</td><td>N/A</td></tr>
<tr><td>Romanian for Romania</td><td>Unknown</td><td>N/A</td><td>N/A</td></tr>
<tr><td>Russian for Russia</td><td>Linguistic validation by Mapi Research Trust[2]</td><td>N/A</td><td>N/A</td></tr>
<tr><td>Serbian for Serbia</td><td>Hajnalka Požar, Medical Faculty, University of Novi Sad, Serbia</td><td>N/A</td><td>N/A</td></tr>
<tr><td>Spanish for Argentina</td><td>Linguistic validation by Mapi Research Trust[2]</td><td>N/A</td><td>N/A</td></tr>
<tr><td>Spanish for Colombia</td><td>Linguistic validation by Mapi Research Trust[2]</td><td>N/A</td><td>N/A</td></tr>
</table>

**Language**	**Translated/adapted by**	**Validation results published**[1]	**Studies using this or another translation in that language**[1]
Spanish for Mexico	Linguistic validation by Mapi Research Trust[2]	N/A	N/A
Spanish for Spain	Ana M Calvo Maroto, University of Valencia, Valencia, Spain<br>Javier Delgado-Lista, Hospital Universitario Reina Sofía, Cordoba, Spain	N/A	Calvo-Maroto AM, Esteve-Taboada JJ, Pérez-Cambrodí RJ, Madrid-Costa D, Cerviño A. Pilot Study on Visual Function and Fundus Autofluorescence Assessment in Diabetic Patients. J Ophthalmol (2016) 2016:1287847. DOI: 10.1155/2016/1287847
Tagalog for the Philippines	Faith Sabellano, Ateneo de Zamboanga University, School of Medicine, Zamboanga City, Philippines	N/A	· Buco CEAM, Buenviaje KAC, Bulan RBC, Cabaña RJL, Cabuhat MKS, Bongar MVV, Macindo JRB. Developing and testing a model of quality of life among chronically-ill, community-dwelling older adults: A structural equation model. Arch Gerontol Geriatr (2018) 78:261-8. DOI: 10.1016/j.archger.2018.07.013<br>· Totesora D, Ramos-Rivera MI, Villegas-Florencio MQ, Reyes-Sia PN. Association of Diabetes-related Emotional Distress with Diabetes Self-care and Glycemic Control among Adult Filipinos with Type 2 Diabetes Mellitus at a Tertiary Hospital in Manila, Philippines. J ASEAN Fed Endocr Soc (2019) 34:189-96. DOI: 10.15605/jafes.034.02.10
Tamil for India	Translation and linguistic validation by Mapi Research Trust[2]	N/A	Manikandaprabu M, Jeyavel S. Type II Diabetic Patients' Illness Perception and Self-care Behaviour: Does Comorbidity make any Difference? Int J Behav Sci (2018) 12:114-24.
Telugu for India	Translation and linguistic validation by Mapi Research Trust[2]	N/A	N/A
Thai for Thailand	Somsak Thojampa, Faculty of Nursing, Naresuan University, Phitsanulok, Thailand & Barbara Mawn, University of	· Thojampa S, Mawn B. Psychometric evaluation of the Thai translation of the Diabetes Self-management Questionnaire in type 2 diabetes. Int J Nurs Sci (2017) 4:236-8. DOI: 10.1016/j.ijnss.2017.06.006	· Thojampaa S, Mawnb B. The moderating effect of social cognitive factors on self-management activities and HbA1c in Thai adults with type-2 diabetes. Int J Nurs Sci (2017) 4:34-7. DOI: 10.1016/j.ijnss.2016.12.006<br>· Boonsatean W, Carlsson A, Dychawy Rosner I, Östman M. Sex-related illness perception and self-management of a Thai type 2 diabetes population: a cross-sectional

Language	Translated/adapted by	Validation results published[1]	Studies using this or another translation in that language[1]
	Massachusetts Lowell, Lowell, MA, US	· Boonsatean W, Carlsson A, Dychawy Rosner I, Östman M. Sex-related illness perception and self-management of a Thai type 2 diabetes population: a cross-sectional descriptive design. BMC Endocr Disord (2018) 18:5. DOI: 10.1186/s12902-017-0229-8	descriptive design. BMC Endocr Disord (2018) 18:5. DOI: 10.1186/s12902-017-0229-8
Turkish for Turkey	Maaike Gerards, University of Amsterdam, the Netherlands<br>Nermin Güdüloğlu et al., Özel Anadolu Sağlık Merkezi Hastanesi, Cumhuriyet Mahallesi, Gebze, Kocaeli, Turkey	Eroğlu N, Sabuncu N. Diyabet Öz yönetim skalası'nın (DÖYS) türk toplumuna uyarlanması: Geçerlik ve güvenirlik çalışması [Adaptation of diabetes self management questionnaire to Turkish society: Validity and reliability study]. J Nurs Sci (2018) 1:1-6. https://dergipark.org.tr/en/pub/hbd/ issue/43003/468294	· Utli H, Doğru BV. The effect of the COVID-19 pandemic on self-management in patients with type 2 diabetics. Prim Care Diabetes (2021) 15:799-805. DOI: 10.1016/j.pcd.2021.07.009<br>· Eroglu N, Sabuncu N. The effect of education given to type 2 diabetic individuals on diabetes self-management and self-efficacy: Randomized controlled trial. Prim Care Diabetes (2021) 15:451-58. DOI: 10.1016/j.pcd.2021.02.011<br>· Sayin Kasar K, Duru Asiret G, Kutmec Yilmaz C, Canlar Ş. The effect of model-based telephone counseling on HbA1c and self-management for individuals with type 2 diabetes: A randomized controlled trial. Prim Care Diabetes (2021) 10:S1751-9918(21)00177-7. DOI: 10.1016/j.pcd.2021.09.005<br>· Kaplan Serin E, Bülbüloğlu S. The Effect of Attitude to Death on Self-Management in Patients With Type 2 Diabetes Mellitus During the COVID-19 Pandemic. Omega (Westport) (2021) 3:302228211020602. DO: 10.1177/00302228211020602
Urdu for India	Translation and linguistic validation by Mapi Research Trust[2]	N/A	N/A
Urdu for Pakistan	Allah Bukhsh, Institute of Pharmaceutical Sciences, University of Veterinary & Animal Sciences, Lahore, Pakistan	Bukhsh A, Lee SWH, Pusparajah P, Schmitt A, Khan TM. Psychometric properties of the Diabetes Self-Management Questionnaire (DSMQ) in Urdu. Health Qual Life Outcomes (2017) 15:200. DOI: 10.1186/s12955-017-0776-8	· Bukhsh A, Khan TM, Nawaz MS, Ahmed HS, Chan KG, Lee L-H, Goh B-H. Association of diabetes-related self-care activities with glycemic control of patients with type 2 diabetes in Pakistan. Patient Prefer Adherence (2018) 12:2377-85. DOI: 10.2147/PPA.S177314

Language	Translated/adapted by	Validation results published[1]	Studies using this or another translation in that language[1]
	Mahnaz Yousaf and Fatima Akram, Institute of Applied Psychology, Queen Mary College Lahore, Pakistan	N/A	· Bukhsh A, Khan TM, Nawaz MS, Ahmed HS, Chan KG, Goh B-H. Association of diabetes knowledge with glycemic control and self-care practices among Pakistani people with type 2 diabetes mellitus. Diabetes Metab Syndr Obes (2019) 12:1409-17. DOI: 10.2147/DMSO.S209711<br>· Bukhsh A, Nawaz MS, Ahmed HS, Khan TM. A randomized controlled study to evaluate the effect of pharmacist-led educational intervention on glycemic control, self-care activities and disease knowledge among type 2 diabetes patients: A consort compliant study protocol. Medicine (Baltimore) (2018) 97:e9847. DOI: 10.1097/MD.0000000000009847<br>· Sayeed KA, Qayyum A, Jamshed F, Gill U, Usama SM, Asghar K, Tahir A. Impact of Diabetes-related Self-management on Glycemic Control in Type II Diabetes Mellitus. Cureus (2020) 12:e7845. DOI: 10.7759/cureus.7845<br>· Akram F, Naz MA. Ego defense mechanisms, medication adherence and self-management of the patients with type 2 diabetes. JPMA (2021) 71:624-8. DOI: 10.47391/JPMA.706
	Linguistic validation by Mapi Research Trust[2]		

[1] Publications displayed as found in literature searches (pubmed/google scholar) in 11/2021.
[2] https://eprovide.mapi-trust.org/instruments/diabetes-self-management-questionnaire; https://eprovide.mapi-trust.org/instruments/diabetes-self-management-questionnaire-revised

Suppl. Table 3. Explanation and rationale of item revisions for the DSMQ-R

**Unaltered items**	Items no. 13 and 16 of the original DSMQ remained unchanged. Item no. 16 of the original DSMQ was moved to position no. 20 in the DSMQ-R.
**Items with minor revisions**	Items no. 1, 2, 4, 5, 10, 11 and 12 were only minimally/slightly altered at which the essential meaning of each statement was not changed (i.e., minor changes of the wording were made or specific terms were added for better understanding or to conform with new developments such as continuous interstitial glucose monitoring (thus the term ‘blood sugar checking’ was replaced with checking ‘glucose levels’). In detail:<br>· Item 1 was revised from ‘I check my blood sugar levels with care and attention.’ to ‘I check my glucose levels with care and attention. corresponding to new measurement technologies (CGM) measuring interstitial glucose rather than blood sugar.<br>· Item 2 was revised from ‘The food I choose to eat makes it easy to achieve optimal blood sugar levels.’ to ‘The foods I choose to eat make it easy for me to achieve good glucose levels.’ corresponding to new measurement technologies (CGM) measuring interstitial glucose rather than blood sugar. The plural form ‘foods’ was used.<br>· Item 4 was revised from ‘I take my diabetes medication (e.g. insulin, tablets) as prescribed.’ to ‘I take my diabetes medication (e.g. insulin, tablets) consistently and reliably.’ to avoid compliance-/adherence-related language.<br>· Item 5 was revised from ‘Occasionally I eat lots of sweets or other foods rich in carbohydrates.’ to ‘I occasionally eat large amounts of sweets or other foods rich in carbohydrates.’, i.e., the order of words was slightly altered and the term ‘large amounts of’ was used instead of ‘lots of’ which is more descriptive.<br>· Item 10 was revised from ‘I do not check my blood sugar levels frequently enough as would be required for achieving good blood glucose control.’ to ‘I do not check my glucose levels frequently enough for achieving good blood glucose control.’ corresponding to new measurement technologies (CGM) measuring interstitial glucose rather than blood sugar.<br>· Item 11 was revised from ‘I avoid physical activity although it would improve my diabetes.’ to ‘I avoid physical activity although it would be good for my diabetes.’ as the extent to which physical activity/exercise can actually improve diabetes (e.g., glucose levels, insulin sensitivity) may depend on diabetes type, treatment regimen and personal glucose variability, while more general positive effects on diabetes and health can more likely be assumed.<br>· Item 12 was revised from ‘I tend to forget to take or skip my diabetes medication (e.g. insulin, tablets).’ to ‘I tend to forget or skip taking my diabetes medication (e.g. insulin, tablets).’, so only a slight revision of the word sequence.
**Items with major revisions**	Items no. 3, 6, 7, 8, 9, 14 and 15 were revised more significantly due to specific considerations; it was tried not to alter the essential meaning of the statement where possible:<br>· In items 3 and 7 regarding contact/cooperation with the diabetes-treating doctor, the concept of ‘keeping doctor’s appointments’ was revised to ‘seeing the doctor regularly’ to avoid compliance-/adherence-related language. In addition, the term ‘diabetes specialist’ was added in parentheses to reflect provision of care by a diabetes team rather than the doctor only. Item 3 was revised from ‘I keep all doctors’ appointments recommended for my diabetes treatment.’ to ‘I regularly see the doctor (/diabetes specialist) regarding my diabetes.’ Item 7 was revised from ‘I tend to avoid diabetes-related doctors’ appointments.’ to ‘I tend to avoid seeing the doctor (/diabetes specialist) regarding my diabetes.’<br>· Item 6 was revised from ‘I record my blood sugar levels regularly (or analyze the value chart with my blood glucose meter).’ to ‘I keep a diary/log of my glucose levels to inform and improve my diabetes management.’ The second part of the

	original item had sometimes led to confusion of respondents suggesting revision. In addition, the sense of recording glucose levels to reflect/better manage one's glucose levels was highlighted, and potential automatic collection of glucose data by CGM was addressed.<br>· Item 8 was revised from 'I do regular physical activity to achieve optimal blood sugar levels.' to 'I am regularly physically active to improve my diabetes and health.' as the extent to which physical activity/exercise can actually improve glucose levels may depend on diabetes type, treatment regimen and personal glucose variability, while more general positive effects on diabetes and health can more likely be assumed.<br>· Item 9 was revised from 'I strictly follow the dietary recommendations given by my doctor or diabetes specialist.' to 'I follow the current dietary recommendations for people with diabetes (e.g. given to me by my doctor or diabetes specialist).' to avoid compliance-/adherence-related language.<br>· Item 14 was revised from 'Regarding my diabetes care, I should see my medical practitioner(s) more often.' to 'Regarding my diabetes, I should see my doctor (/diabetes specialist) more often.', so only a slight revision of the wording regarding diabetes and doctors/healthcare professionals.<br>· Item 15 was revised from 'I tend to skip planned physical activity.' to 'I am less physically active than would be good for my diabetes.' since the original item was not really related to diabetes self-management constituting a possible limitation; and 'less active' is more dimensional and less judgmental than 'skipping' intended exercise.
**Newly added items**	In addition to the above amendments, several new items were added. Four items (no. 16, 17, 18, 19) were added to expand on some aspects which were felt were not optimally addressed in the original version, resulting in a new total of 20 items:<br>· Item 16 of the DSMQ-R stating 'I could improve my diabetes self-care considerably.' was added to request an overall evaluation of one's diabetes self-management activities, comparably to the original version's item 'My diabetes self-care is poor', however, less negative and judgmental (i.e., less prone to social desirability bias).<br>· Item 17 stating 'I estimate the carbohydrate content of my meals/foods (to improve my diabetes control).' was added to cover the important aspect of carbohydrate estimation which was not included in the original version.<br>· Item 18 stating 'I eat without regard to my diabetes.' was added as a part of the eating behavior scale formerly consisting of four items on choosing foods facilitating glucose control (item 2); eating high-carb foods (item 5); following dietary recommendations (item 9); and potential binging (item 13). It constitutes a contrary to item 2 i.e. is a negatively-keyed item on choosing proper foods for easier diabetes management.<br>· Item 19 stating 'I check and discuss my diabetes treatment with the doctor (/diabetes specialist) regularly.' was added to address goal-oriented collaboration with healthcare professionals for optimizing treatment modalities and procedures which was felt was not satisfactorily covered by the original version.; this constitutes a basis for choosing optimal medical treatments and contributes to treatment motivation and more optimal self-management.<br>Further seven items (no. 21 to 27) were newly added in the revised DSMQ to cover specific self-management behaviors of relevance where intensive insulin treatment is used. These new items are separated and characterized as optional in the corresponding additional instruction, so respondents are asked to respond to these items if they use rapid acting insulin to be inject before meals only. The optional items assess the following activities:

	· Item 21 stating 'I check my glucose levels before each meal.' Was added as this constitutes a basic requirement for adapting insulin doses properly.<br>· Item 22 stating 'I adjust my insulin doses to the carbohydrate content of my meals' was added since proper adaption of insulin doses to the carbohydrate content of the meals is essential for achieving optimal glycemic outcome and avoiding hypo- or hyperglycemia.<br>· Item 23 stating 'I adjust the timing of my insulin injections to the start of my meals.' was added since proper timing of insulin injection to food intake is essential for achieving optimal glycemic outcome and avoiding hypo- or hyperglycemia.<br>· Item 24 stating 'I adjust my insulin doses according to the current glucose levels and preceding or planned activities.' was added since proper adjusting of insulin doses to current glucose levels and activities is essential for achieving optimal glycemic outcome and avoiding hypo- or hyperglycemia.<br>· Item 25 stating 'I correct elevated glucose levels consistently whenever necessary.' was added to cover doing corrective insulin injections as an essential activity for achieving optimal glycemic outcome and reducing hyperglycemia.<br>· Item 26 stating 'I carry fast carbohydrates to enable quick treatment of low blood glucose.' was added since carrying fast carbs is a requirement for correcting low glucose levels and avoiding hypoglycemia.<br>· Item 26 stating 'In case of low blood glucose, I take appropriate amounts of carbohydrates to avoid causing high blood glucose.' was added since taking overly large amounts of carbs during hypoglycemic attacks can easily happen whether intentionally or not (e.g., by mistake in a state of reduced consciousness, due to hunger feeling, due to worry of taking too little and staying low with risk of losing consciousness) and leads to suboptimal glycemic outcome. It can also lead to frustration possibly impacting on self-management motivation.

Suppl. Table 4. Ecological momentary assessment (EMA) items regarding diabetes self-management requested in the DIA-LINK studies

DIA-LINK1 Study EMA items	
1) How much have you felt overwhelmed by your diabetes treatment today?<br>2) How much have you felt guilty when neglecting your diabetes treatment today?	· The questions were asked daily in the evenings over 17 consecutive days using a smartphone app.<br>· Response options were from 0–'not at all' to 10–'very much'.<br>· Item scores were averaged to a mean score for correlation analysis with the DSMQ-R.
DIA-LINK2 Study EMA items	
1) How much have you felt overwhelmed by your diabetes treatment today?<br>2) How much have you felt guilty when neglecting your diabetes treatment today?<br>3) How much do you think has your diet today promoted good glucose control?<br>4) How much do you think your diet today is beneficial to your weight?<br>5) How well have you been able to implement your medical diabetes treatment (insulin, tablets) today?<br>6) How much do you think your physical activity behavior today has contributed to good diabetes management?<br>7) How do you rate the quality of your self-management behavior overall today?	· The questions were asked daily in the evenings over 17 consecutive days using a smartphone app.<br>· Response options were from 0–'not at all' to 10–'very much'.<br>· Item scores were averaged to a mean score for correlation analysis with the DSMQ-R.

Suppl. Table 5. Internal reliability coefficients compared for scales including the new items versus original items only

			T1D			T2D		
**Study**	**Scale**	***N* of items in scale**	Original scale	Revised scale	Revised scale+	Original scale	Revised scale	Revised scale+
Study 1 *n*=259 with T1D[1] *n*=87 with T2D[1]	*Total scale*	16	0.90 (0.90)			0.86 (0.86)		
		20		0.92 (0.92)			0.87 (0.87)	
		27			0.94 (0.95)			0.90 (0.90)
	Eating behavior	4	0.80 (0.80)			0.74 (0.75)		
		6		0.82 (0.83)			0.75 (0.75)	
	Cooperation with diabetes team	3	0.69 (0.69)			0.53 (0.53)		
		4		0.75 (0.76)			0.60 (0.60)	
Study 2 *n*=126 with T1D[1] *n*=40 with T2D[1]	*Total scale*	16	0.89 (0.89)			0.86 (0.86)		
		20		0.91 (0.91)			0.86 (0.85)	
		27			0.93 (0.93)			0.90 (0.90)
	Eating behavior	4	0.76 (0.75)			0.70 (0.70)		
		6		0.79 (0.80)			0.68 (0.69)	
	Cooperation with diabetes team	3	0.81 (0.82)			0.73 (0.73)		
		4		0.85 (0.86)			0.79 (0.78)	

Study 3<br>*n*=188 with T1D[1]<br>*n*=75 with T2D[1]	*Total scale*	16	0.85 (0.85)			0.86 (0.86)		
		20		0.88 (0.88)			0.88 (0.88)	
		27			0.91 (0.91)			0.90 (0.91)
	Eating behavior	4	0.67 (0.68)			0.72 (0.72)		
		6		0.71 (0.72)			0.78 (0.78)	
	Cooperation with diabetes team	3	0.77 (0.78)			0.65 (0.67)		
		4		0.80 (0.80)			0.66 (0.66)	
Study 4<br>*n*=203 with T1D[1]	*Total scale*	16	0.86 (0.86)			n/a		
		20		0.88 (0.88)			n/a	
		27			0.91 (0.91)			n/a
	Eating behavior	4	0.70 (0.70)			n/a		
		6		0.74 (0.75)			n/a	
	Cooperation with diabetes team	3	0.76 (0.76)			n/a		
		4		0.83 (0.83)			n/a	
Study 5<br>*n*=118 with T2D[1]	*Total scale*	16	n/a			0.88 (0.88)		
		20		n/a			0.90 (0.90)	
		27			n/a			0.92 (0.92)
	Eating behavior	4	n/a			0.78 (0.77)		

	6		n/a		0.80 (0.80)
Cooperation with diabetes team	3	n/a		0.63 (0.64)	
	4		n/a		0.75 (0.75)

Notes: Displayed are Cronbach's α (McDonald's ω) for each DSMQ-R scale stratified by study and diabetes type.
MDI, multiple daily (insulin) injections; n/a, not available; T1D, type 1 diabetes; T2D, type 2 diabetes.
[1] Participants who did not respond to all 27 items of the DSMQ-R (incl. items 21–27) were excluded to warrant full scale comparisons; i.e., for T2D, persons without bolus insulin and multiple daily insulin injections (MDI) were excluded.

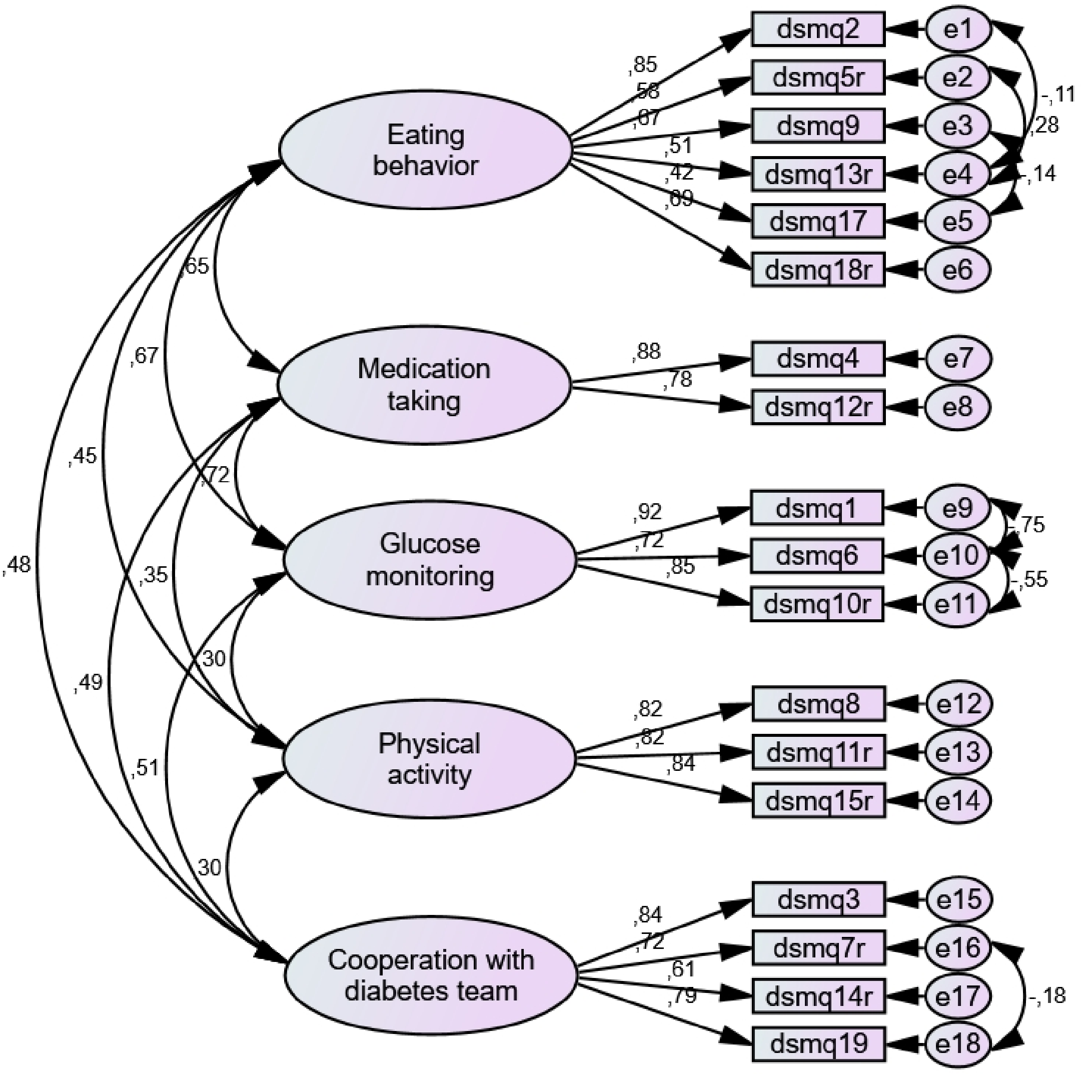

Suppl. Figure 1. Confirmatory factor analysis testing a five-factor model representing the DSMQ-R subscales on people with T1D

Notes: Shown is the ‘fitted’ model with six significant error term correlations modelled.*
Data base: *N*=858 (data from all studies aggregated).
Model fit: CFI=0.961, TLI=0.950, SRMR=0.039, RMSEA=0.052 (90% CI 0.046–0.058).
All factor loadings significant with *p*<0.001. All factor correlations significant with *p*<0.001.
Correlations between error weights significant with *p*≤0.011.
* Model fit before modelling error term correlations was: CFI=0.939, TLI=0.926, SRMR=0.050, RMSEA=0.063 (90% CI 0.058–0.068).

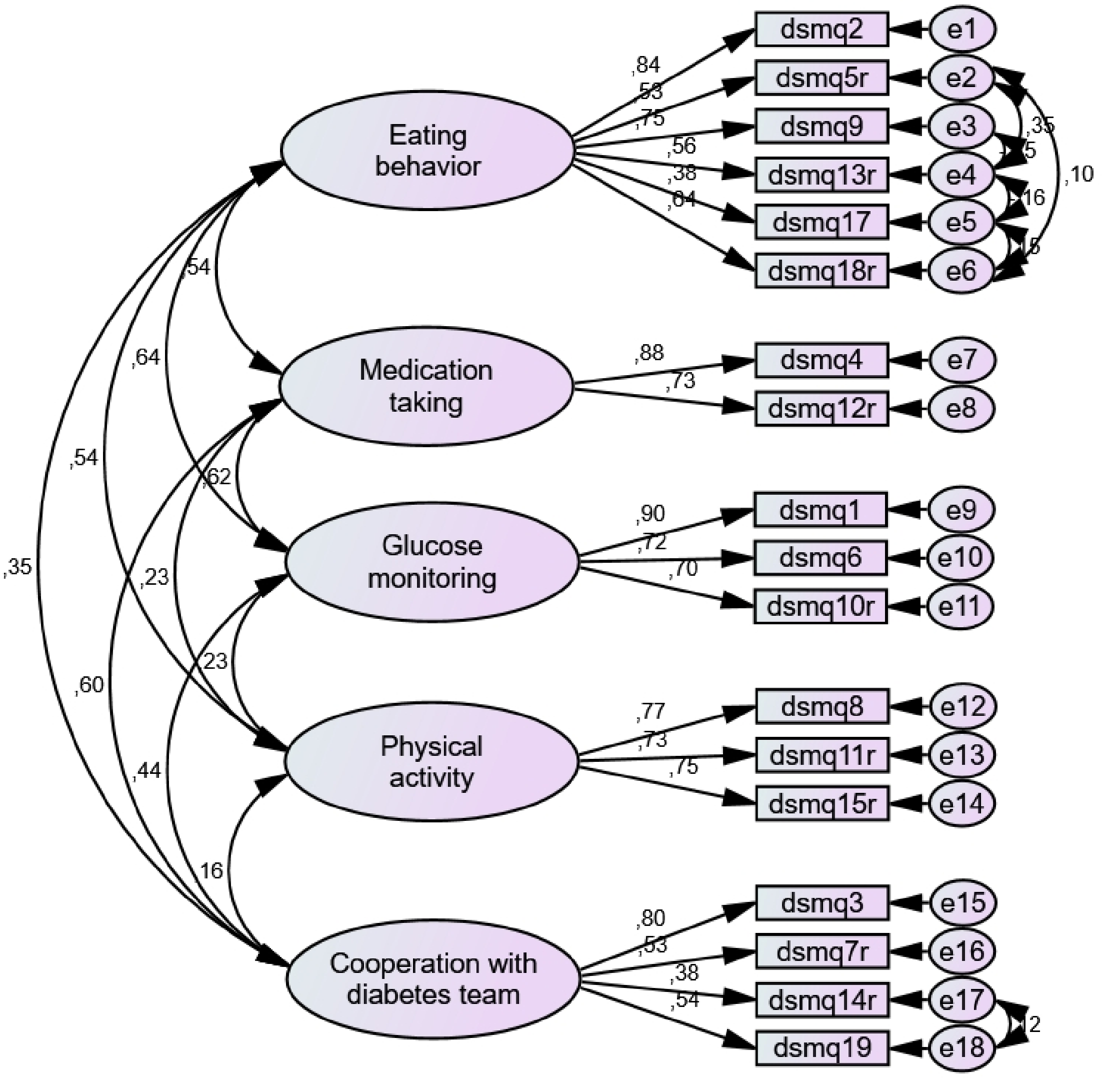

Suppl. Figure 2. Confirmatory factor analysis testing a five-factor model representing the five subscales on people with T2D

Notes: Shown is the ‘fitted’ model with six significant error term correlations modelled.*
Data base: *N*=603 (data from all studies aggregated).
Model fit: CFI=0.953, TLI=0.940, SRMR=0.039, RMSEA=0.036 (90% CI 0.043–0.057).
All factor loadings significant with $p<0.001$. All factor correlations significant with $p\leq0.004$.
Correlations between error weights significant with $p\leq0.012$.
* Model fit before modelling error term correlations was: CFI=0.920, TLI=0.903, SRMR=0.043, RMSEA=0.064 (90% CI 0.057–0.070).

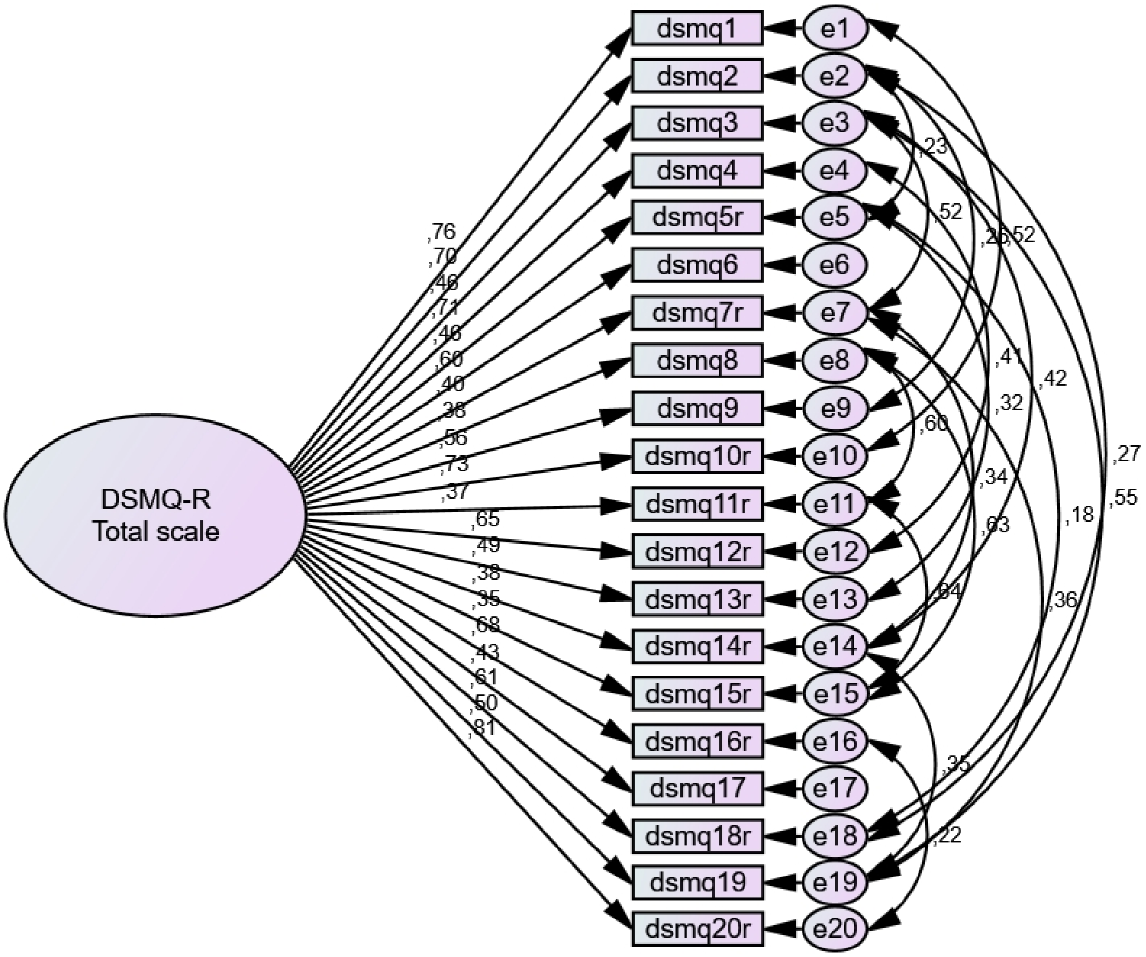

Suppl. Figure 3. Confirmatory factor analysis testing a one-factor model representing the 20-item total scale on people with T1D

Notes: Shown is the ‘fitted’ model with 17 significant error term correlations modelled.*
Data base: *N*=858 (data from all studies aggregated).
Model fit: CFI=0.947, TLI=0.934, SRMR=0.041, RMSEA=0.058 (90% CI 0.053–0.063).
All factor loadings significant with *p*<0.001. All correlations between error weights significant with *p*<0.001.
* Model fit before modelling error term correlations was: CFI=0.654, TLI=0.613, SRMR=0.099, RMSEA=0.141 (90% CI 0.137–0.145). Correlations were modelled only for sensible variable associations with high modification indices (>20).

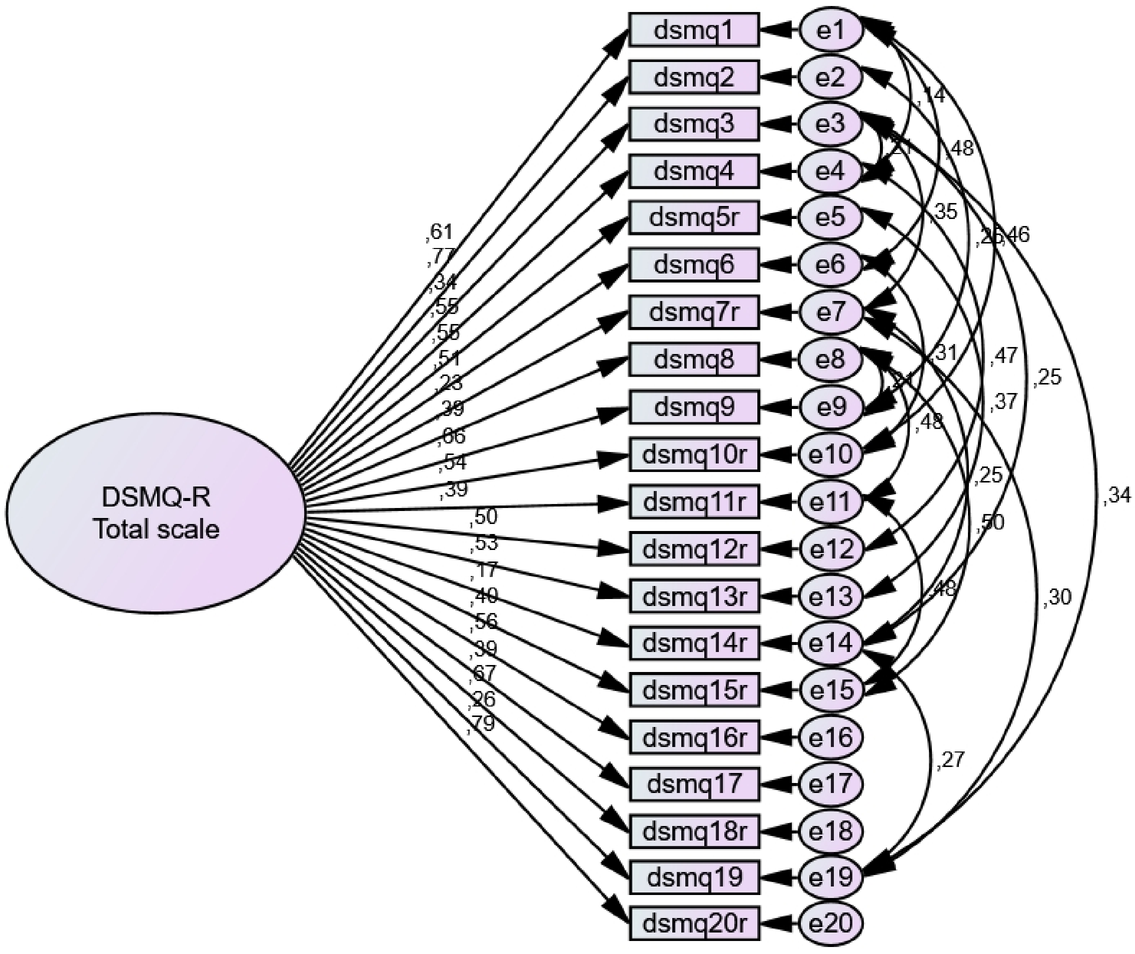

Suppl. Figure 4. Confirmatory factor analysis testing a one-factor model representing the 20-item total scale on people with T2D

Notes: Shown is the 'fitted' model with 18 significant error term correlations modelled.*
Data base: *N*=603 (data from all studies aggregated).
Model fit: CFI=0.936, TLI=0.920, SRMR=0.053, RMSEA=0.056 (90% CI 0.050–0.062).
All factor loadings significant with $p<0.001$. All correlations between error weights significant with $p<0.001$.
* Model fit before modelling error term correlations was: CFI=0.647, TLI=0.606, SRMR=0.097, RMSEA=0.125 (90% CI 0.119–0.130). Correlations were modelled only for sensible variable associations with high modification indices (>20).

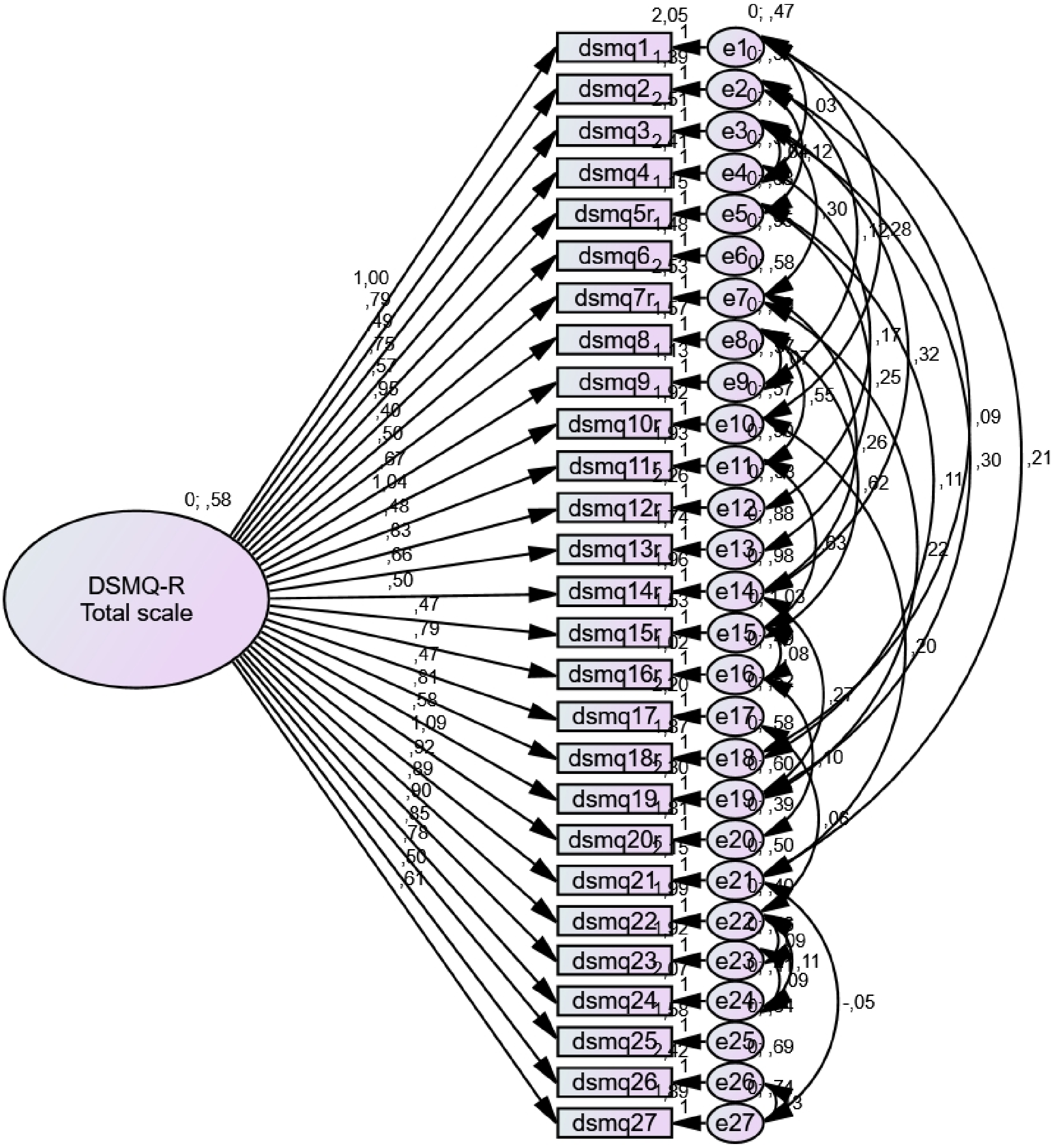

Suppl. Figure 5. Confirmatory factor analysis testing a one-factor model representing the 27-item total scale on people with T1D

Notes: Shown is the ‘fitted’ model with 29 significant error term correlations modelled.*
Data base: *N*=778 (data from all studies aggregated).
Model fit: CFI=0.946, TLI=0.936, SRMR=0.040, RMSEA=0.050 (90% CI 0.047–0.054).
All factor loadings significant with $p<0.001$. All correlations between error weights significant with $p\leq0.008$.
* Model fit before modelling error term correlations was: CFI=0.697, TLI=0.672, SRMR=0.084, RMSEA=0.114 (90% CI 0.111–0.118). Correlations were modelled only for sensible variable associations with high modification indices (>20).

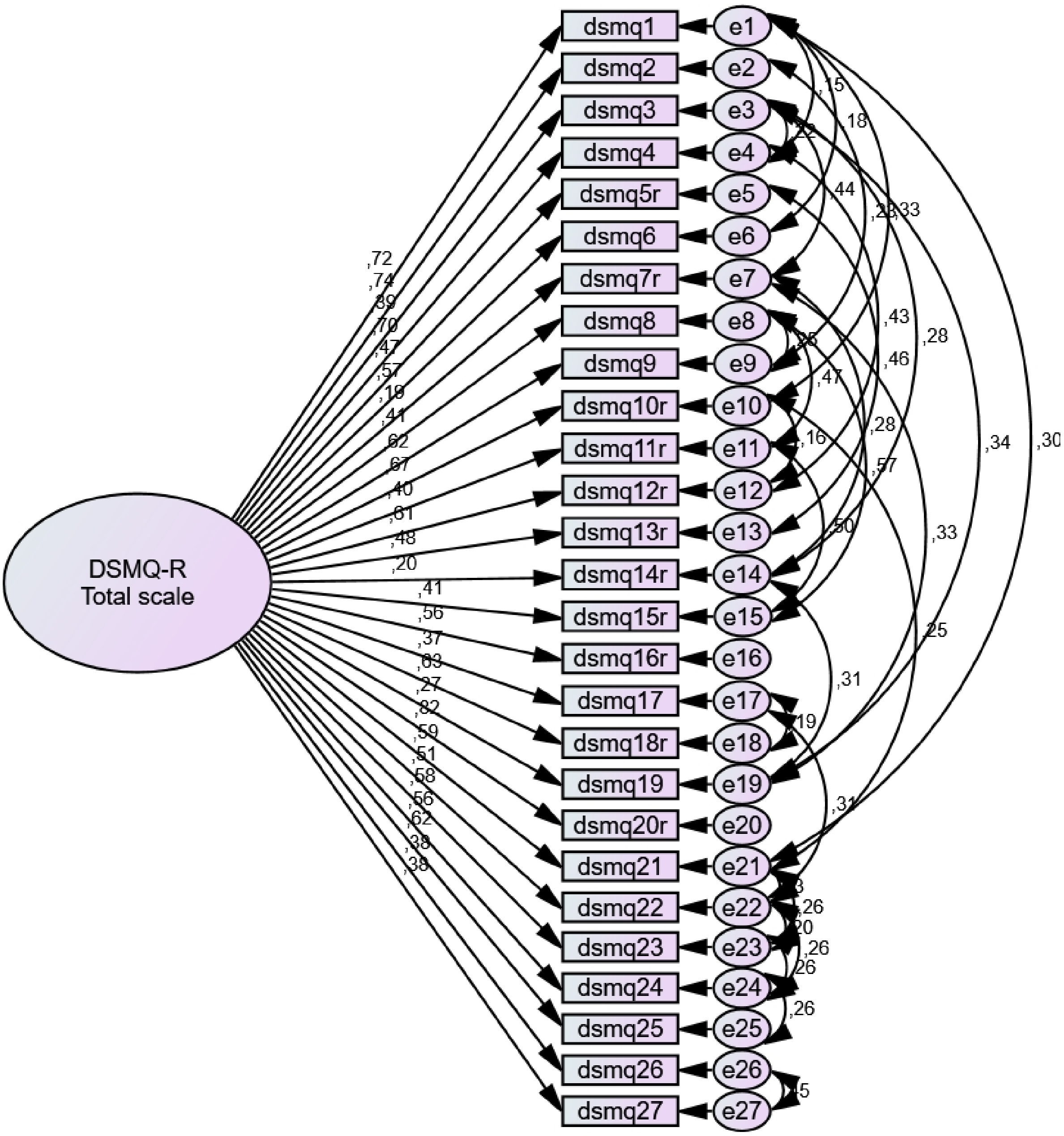

Suppl. Figure 6. Confirmatory factor analysis testing a one-factor model representing the 27-item total scale on people with T2D

Notes: Shown is the 'fitted' model with 29 significant error term correlations modelled.*
Data base: *N*=325 (data from all studies aggregated).
Model fit: CFI=0.922, TLI=0.907, SRMR=0.056, RMSEA=0.055 (90% CI 0.049–0.062).
All factor loadings significant with *p*≤0.001. All correlations between error weights significant with *p*≤0.001.
* Model fit before modelling error term correlations was: CFI=0.655, TLI=0.626, SRMR=0.091, RMSEA=0.111 (90% CI 0.106–0.116). Correlations were modelled only for sensible variable associations with high modification indices (>20).
